# Evaluating the use of whole-genome sequencing and Sanger emm typing in guiding the identification and public health management of invasive group A Streptococcus clusters and outbreaks in England

**DOI:** 10.1099/jmm.0.002146

**Published:** 2026-03-25

**Authors:** Kathrin Loosli, Juliana Coelho, Kartyk Moganeradj, Derren Ready, Charles R. Beck, Nicola Love

**Affiliations:** 1 Evaluation and Epidemiological Science Division, Chief Scientific Officers’ Group, UKHSA, London, UK; 2 Staphylococcus & Streptococcus Reference Service, Antimicrobial Resistance and Healthcare Associated Infections, Chief Scientific Officers’ Group, UKHSA, London, UK; 3 South West Field Services, Health Protection Operations,Chief Medical Advisor’s Group, UKHSA, Bristol, UK; 4 National Institute of Health and Care Research Health Protection Research Unit in Evaluation and Behavioural Science, Bristol, UK

**Keywords:** bacterial typing techniques, evaluation, infection prevention and control, invasive group A *Streptococcus* (iGAS), public health practice, public health surveillance, streptococcal infection, *Streptococcus pyogenes*, whole-genome sequencing (WGS)

## Abstract

**Introduction.** Sanger sequencing-based *emm* typing has traditionally been used in England to assess case relatedness when managing invasive group A *Streptococcus* (iGAS) outbreaks.

**Gap statement.** While timely, *emm* typing can lack resolution. Whole-genome sequencing (WGS), offering greater strain discrimination, is increasingly used, but its comparative value for public health response has not been assessed.

**Aim.** This study evaluates the added value of WGS over Sanger *emm* typing for group A *Streptococcus* (GAS) surveillance and outbreak detection, investigation and management in England.

**Methodology.** The evaluation followed the Pathogen Genomics in Public Health Surveillance Evaluation (PG-PHASE) framework (full description in Appendix 1, Table S1). Interviews with laboratory staff and bioinformaticians, and a survey of UK Health Security Agency (UKHSA) health protection and epidemiology iGAS leads, provided insights into *emm* typing and WGS data use, utility, acceptability and appropriateness. Typing data from outbreak isolates collected between February 2018 and February 2024 were analysed to evaluate the concordance between Sanger *emm* typing and WGS-based single-nucleotide polymorphism (SNP) clusters. WGS strain diversity and SNP distances within *emm* types were assessed, using a five-SNP threshold for WGS clusters. The number of cases excluded from outbreaks by *emm* typing vs. WGS was compared.

**Results.** Laboratory staff indicated that transitioning from a Sanger- to a WGS-based GAS typing service would result in more granular information to inform public health action. Survey participants acknowledged WGS benefits for relatedness resolution but raised concerns about longer turnaround times (a maximum of 14 vs. 5 days for WGS and the Sanger method, respectively) and highlighted the need for training in interpreting WGS data. Suggestions included more standardized, regular, cumulative WGS reports to improve clarity and usefulness. We analysed 178 epidemiologically linked GAS outbreaks, including 1,142 isolates. *Emm* typing excluded 217 unrelated isolates from 58 outbreaks, while WGS excluded a further 224 isolates (*n*=44 total) from 114 outbreaks.

**Conclusion.** WGS improves outbreak management, particularly for protracted outbreaks and those with common *emm* types. WGS better automates and integrates reference laboratory services; however, current turnaround times reduce its benefits for immediate public health action. Our findings suggest that strengthening the timeliness of WGS reporting, co-designing reporting processes with data users and providing training in data interpretation will enhance WGS utility.

## Data Summary

Data cannot be shared publicly because the data analysed were collected as part of a service evaluation, and consent has not been obtained to share these data beyond the UK Health Security Agency (UKHSA). Any requests for data should be directed to the corresponding author and will be managed in line with UKHSA policies and procedures (see: https://www.gov.uk/government/publications/accessing-ukhsa-protected-data/accessing-ukhsa-protected-data).

## Introduction

Group A *Streptococcus* (GAS), also known as *Streptococcus pyogenes*, is a highly pathogenic Gram-positive bacterium, which results in a range of presentations from mild, self-limiting skin and throat conditions to severe invasive infections [1,2]. The burden of GAS in England is substantial. Surveillance of GAS (both invasive and non-invasive infections) showed an increase from 2.4 to 4.8 cases per 100,000 population between 2014 and 2018 [3]. After declining during the Coronovirus Disease 2019 (COVID-19) pandemic, invasive GAS (iGAS) cases rose sharply in 2022/2023, surpassing pre-pandemic levels [4,5]. Between weeks 37 and 48 of 2022, UK Health Security Agency (UKHSA) received 772 iGAS notifications, and a notable increase in severe morbidity among children was observed (26.1% of all cases), including serious respiratory complications [5]. Since then, case numbers have returned to levels consistent with those observed before the pandemic [6].

Cases of iGAS are defined in UKHSA guidelines as cases with the isolation of GAS from a normally sterile site, such as blood and/or cerebrospinal fluid, or cases with a severe clinical presentation where GAS has been isolated from a normally non-sterile site [1]. iGAS is characterized by rapid onset and fast progression, and the dominant lineages currently present in England have demonstrated high 30-day case fatality rates, ranging from 10.5% to 24.4% in the pre-COVID-19 period [7]. Population groups at highest risk of developing invasive infections include the elderly (particularly those aged 75 years or over), young children, neonates, postpartum women in the neonatal period, people who experience homelessness, people who inject drugs, people with skin lesions and those with comorbidities such as diabetes, cardiovascular disease, immunosuppression or with influenza or recent chickenpox [1,2, 8, 9]. Demographic characteristics associated with higher risk of iGAS include being of non-white ethnicity, male sex or belonging to a disadvantaged population group [1,2, 10], highlighting health inequalities and a disproportionate disease burden. There is a need to rapidly investigate clusters or outbreaks of iGAS to identify potential sources of infection and limit morbidity and mortality to protect these vulnerable populations [2]. Sanger sequencing-based *emm* typing, which differentiates GAS strains based on the sequence of the *emm* gene, a key virulence factor, has traditionally been used as the ‘gold standard’ typing method in England. While over 200 *emm* types exist globally, in 2022/2023, 62% of typed iGAS isolates from the UK were from five *emm* types, with *emm*1 accounting for 24% of isolates [11].

While the majority of iGAS infections in England are sporadic, outbreaks have been detected in institutional settings including long-term care facilities, schools and nurseries, or associated with community healthcare providers [1]. However, outbreaks may be under-recognized due to the limitations of Sanger *emm* typing in recognizing genetic links, particularly where common *emm* types are involved. In such cases, the presence of the same *emm* gene across multiple isolates provides insufficient resolution to distinguish between unrelated cases and those that may be part of a transmission cluster, meaning that seemingly sporadic infections may in fact represent smaller, unidentified clusters [9]. The limited resolution of *emm* typing can also complicate transmission pathway identification and delay control measures.

Whole-genome sequencing (WGS) offers greater typing precision than *emm* gene typing through single-nucleotide polymorphism (SNP) analysis. Studies have highlighted the enhanced performance of WGS for both GAS outbreak investigation and surveillance [12,17], with WGS being particularly powerful in identifying associations between cases without apparent epidemiological links [15]. UKHSA has run WGS in parallel with Sanger *emm* typing on an ad hoc basis since 2018 and then on all iGAS and outbreak-related non-iGAS isolates from September 2022. WGS-based *emm* typing was undertaken as part of the WGS pipeline, but results were not reported to service users because those obtained from Sanger sequencing were routinely reported. The only WGS data provided to end users at the time of this study were SNP analyses conducted on request only, predominantly to support outbreak activities.

Despite the increasing use of WGS, its added value over established typing approaches and its fitness for purpose for routine GAS outbreak detection, investigation and public health management remained unclear, particularly in terms of how WGS-derived outputs could be interpreted, integrated into public health decision-making and used to inform timely action. We therefore conducted an evaluation of Sanger sequencing-based *emm* typing and WGS to assess the added public health value of WGS in outbreak contexts and to ensure that WGS is applied in ways that most effectively support public health action. An assessment of costs, health economic endpoints or the utility of WGS for routine surveillance of GAS was beyond the scope of this evaluation.

## Methods

The evaluation used a mixed-method approach and was structured using the Pathogen Genomics in Public Health Surveillance Evaluation (PG-PHASE) framework, which is used to evaluate the implementation of WGS in public health settings [18]. The framework explores three phases relating to the implementation of WGS: (i) the pre-analysis and analysis of typing data, (ii) reporting and communication of typing data, and (iii) implementation of typing in public health practice [18].

### Stakeholder interviews

Semi-structured interviews were conducted with reference laboratory staff and bioinformaticians undertaking GAS typing and WGS analysis in the *Staphylococcus* and *Streptococcus* Reference Section (SSRS), UKHSA. Interviews were conducted using Microsoft Teams in April 2024. All interviews were conducted one-on-one, with the exception of one interview conducted with two people in the same job role. The interview schedule covered the interviewees’ role in the GAS typing workflow (including sample preparation, sequencing and data analysis stages for Sanger *emm* typing and WGS), strengths and limitations of each typing method and potential future developments (Appendix 7, available in the online Supplementary Material). Interviews were recorded and automatically transcribed in Microsoft Teams with transcript, which was checked against the video recording for accuracy before being thematically coded in NVivo 14 (version 14.23.1). The thematic analysis approach involved initial familiarization with the data, iterative coding and the establishment of themes [19]. Codes were arranged into a project map (Appendix 2, Figure S1).

### Service user survey

An online survey was created using SelectSurvey (SelectSurvey.NET v5.065.003, ClassApps.com, Apollo Beach, FL, USA) and disseminated by email to service users, including iGAS leads within regional Health Protection Teams (HPTs) and Field Epidemiology teams [Field Service (FS)]. Closed and open-ended questions were used to capture feedback on typing data availability and use for surveillance, outbreak management and public health action. All WGS-related survey questions focused on SNP analysis, as it was the only WGS-based data available at the time. Information on the utility, timeliness and appropriateness of the typing data provided was captured, and stakeholders were asked for feedback on improving data reporting (Appendix 8). Responses were analysed descriptively using R statistical software (version 4.4.0) in R Studio (version 2024.04.1.0). Questions were not mandatory, and conditional questions were used; therefore, the denominator was question-specific and determined using the number of unique responses. Narrative responses were either grouped into categories and analysed by thematic coding as described above or analysed quantitatively by counting response frequency.

### Reference laboratory typing data

*Emm* typing data were received from the reference laboratory for a subset of GAS isolates reported between May 2015 and February 2024, which were epidemiologically linked in outbreak clusters. Isolate-level metadata included patient name, age, postcode, diagnosis, isolation site (sterile or non-sterile) and laboratory receipt date. Isolates within the same epidemiologically linked outbreak were identified by a common outbreak idenfitication number. For each outbreak, the location, setting and main *emm* type were provided. SNP analysis data were also provided for isolates within the subset that had undergone WGS sequencing between February 2018 and February 2024. The year of the earliest isolate, derived from reference laboratory specimen reception date, was used to date epidemiologically linked clusters. Where outbreak setting information was missing, it was inferred from the outbreak description (e.g. mentions of hospitals, prisons or care homes). If no clear setting could be determined, it was recorded as ‘unknown’.

Isolates from the same case within the same outbreak were deduplicated to one isolate per case per outbreak if all isolates were of the same *emm* type. Where a case had isolates of more than one *emm* type within the same outbreak, one isolate of each *emm* type was retained. Cases associated with more than one outbreak retained one isolate per outbreak, even if isolates were the same *emm* type. During deduplication, priority was given to isolates that had SNP information available. For each episode, the earliest isolate with SNP information was retained. If no SNP results were available, the earliest isolate overall was retained.

**Fig. 1.**
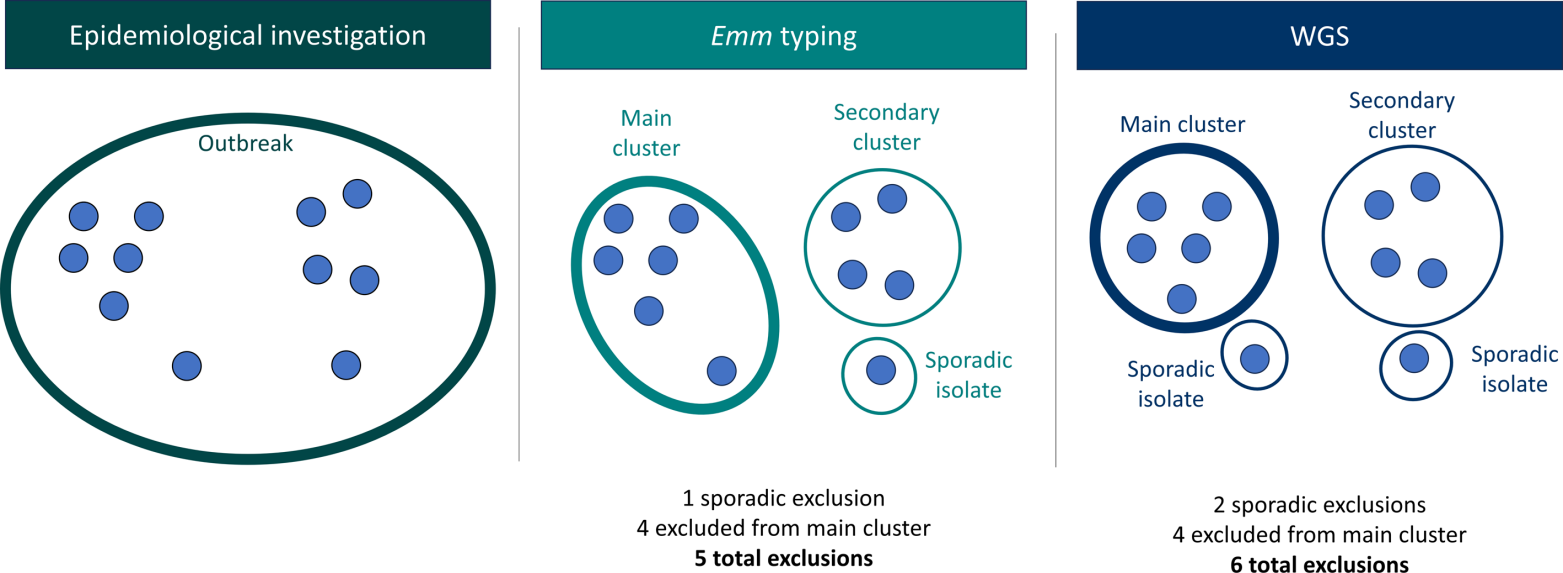
Methodology to calculate the number of excluded cases from an epidemiologically linked outbreak. The figure shows the process of excluding isolates from a defined epidemiologically linked outbreak (left) using *emm* typing (middle) and WGS (right). Total exclusions are calculated by counting all isolates that were not associated with the main (largest) cluster. Sporadic exclusions include isolates that did not cluster with any other isolates in the same outbreak by either *emm* typing or WGS. Isolates that clustered in a secondary cluster were also counted as excluded from the main outbreak cluster.

A 0–5 SNP threshold was used to define WGS clusters, reflecting UKHSA practice and balancing the typical GAS mutation rate of 1–3 SNPs per year with variability across *emm* types, some of which are more clonal than others [20,22]. Genetic diversity, defined as the number of WGS clusters and SNP levels within each *emm* type and epidemiologically related *emm* cluster, was assessed descriptively. The number of cases excluded from epidemiologically linked clusters by *emm* typing or WGS was determined by calculating the number of cases that did not associate with the largest *emm*/WGS cluster within the epidemiologically linked outbreak (Fig. 1). Exclusions were categorized either as sporadic (a single isolate of a different *emm* type/WGS profile to the main cluster) or as a secondary cluster (multiple isolates clustering in a smaller cluster than the main *emm*/WGS cluster). This distinction is important because cases in a secondary cluster may be investigated as a separate incident. In outbreaks where the two largest *emm* or WGS clusters were the same size, the designation of 'main’ cluster – the cluster considered part of the outbreak vs. the cluster considered excluded – was assigned at random. This had no impact on the analysis since the clusters were identical in size, making the assignment of one as part of the outbreak and the other as an excluded secondary cluster analytically inconsequential. Definitions of terms related to this analysis are provided in Table S2, Appendix 3.

## Results

### Pre-analysis and analysis of typing data

At the time of evaluation, the published maximum turnaround time for Sanger-based *emm* typing was 8 days [23], but most isolates were typed within 3–5 days of reception.

**Fig. 2.**
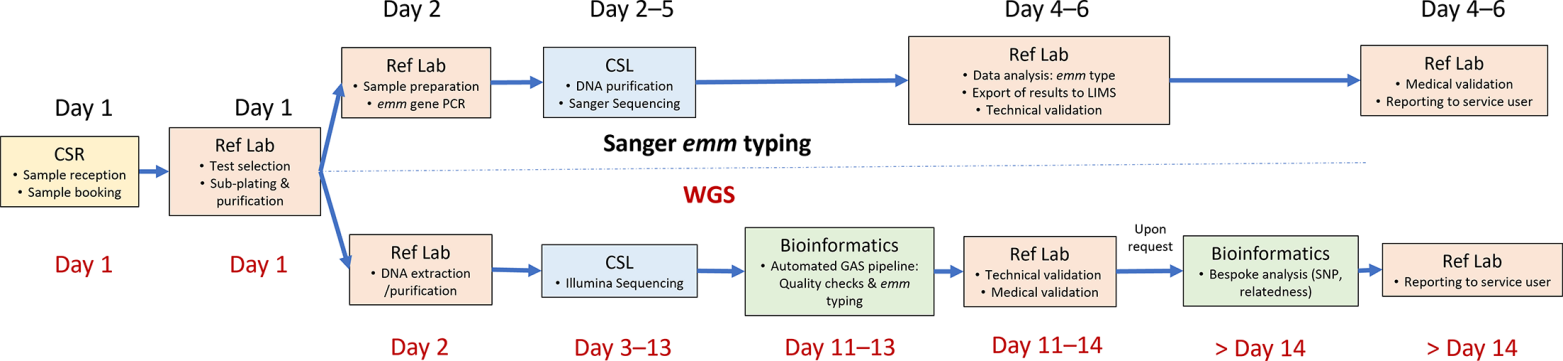
Diagram of workflows for Sanger *emm* typing and WGS. Boxes indicate locations where isolates/typing data are processed, and associated actions are listed for both Sanger *emm* typing (top) and WGS (bottom). Days after reception at which the various steps happen are indicated for both workflows separately (*emm* typing in black text, WGS in red text). CSL, Colindale Sequencing Laboratory; CSR, Central Specimen Reception; LIMS = Laboraotry Information Management System; Ref Lab = Reference Laboratory

WGS-based *emm* typing results were generated within 10–14 days. These turnaround times reflect routine processing timelines at the central UKHSA sequencing laboratory, which receives and processes a high volume of isolates from across the agency. The timeline for bespoke SNP phylogenetic analyses was dependent on reference laboratory bioinformatician capacity but generally took longer than the 10–14 days needed for WGS-based *emm* typing. Further details of laboratory workflows can be found in Fig. 2 and Appendix 4.

#### Transitioning from a Sanger-based to a WGS-based typing service

Reference laboratory staff (*n*=6) were asked in interviews for their views on transitioning to a WGS-only-based GAS typing service for both *emm* typing and SNP analysis. Participants noted advantages of WGS, including the automated workflow, which would reduce human error, and the better integration with other existing WGS-based typing services. Staff also recognized that WGS would generate further insights beyond *emm* typing, such as phylogenetic analyses and the tracking of antimicrobial resistance (AMR) genes, which would improve pathogen characterization and surveillance. As one participant stated, ‘*it’s going to be less work in the beginning, more information at the end*’.

Drawbacks of a WGS-based service were acknowledged, such as longer turnaround times and the need to train more staff on WGS workflows, but SRSS staff favoured WGS over Sanger *emm* typing as it was seen to better meet current and future needs.

*WGS, in my opinion, is hugely beneficial for the customer because it really helps them in outbreak management and outbreak investigations. It gives them much more data, much more information. […] So, if the turnaround time is within a reasonable time, then I'd definitely be all for WGS. But the turnaround time is the key*.

-Reference laboratory member

### Reporting and communication of typing data

#### Reporting processes

**Fig. 3.**
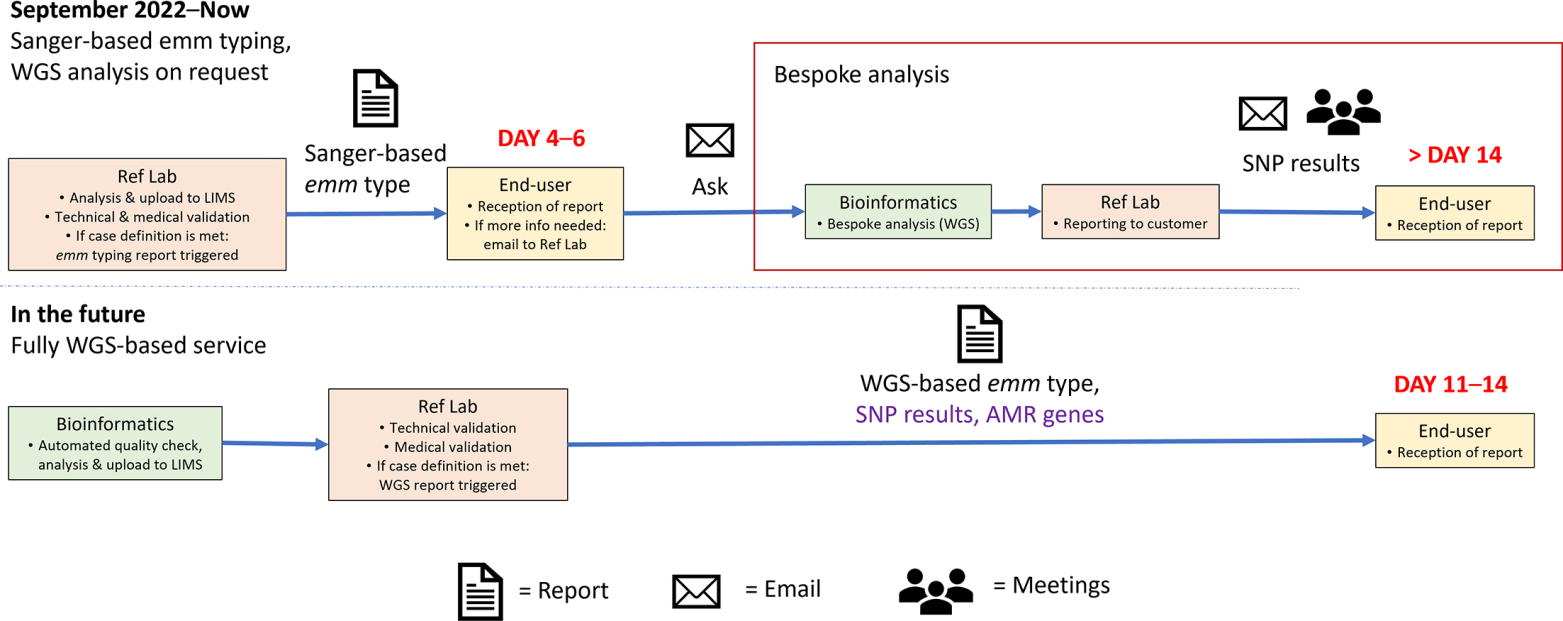
Diagram of reporting processes for *emm* typing data within UKHSA. Reporting mechanisms from 2022 to date (top) and the proposed future mechanism (bottom) are shown. Boxes indicate locations through which the isolates/data travel and associated actions are listed. The number of days, indicated in red, indicates the time elapsed from specimen receipt at SSRS to when reports are received by UKHSA service users. The red box indicates bespoke processes available on request. Text in purple indicates outputs that are still under development*. *LIMS = Laboratorty Information Management System; Ref Lab = Reference Laboratory

Sanger *emm* typing results were reported to UKHSA staff working in regional epidemiology and HPTs (defined as service users) via an automated, online, isolate-level report. While WGS-based automatic *emm* typing reports were still under development and not yet available, users could request bespoke WGS-based SNP analysis to support outbreak activities. Further details on reporting of Sanger and WGS typing results can be found in Fig. 3 and Appendix 5.

#### Service user perceptions of data utility, timeliness and suitability

Fifteen survey responses were received from 6 out of 9 (55.5%) regional epidemiology teams and 9 out of 15 (53.3%) regional HPTs (one joint epidemiology/HPT response) and one national team, representing responses from 8/9 English regions. Response rates varied by question, with free-text questions generally poorly completed.

**Fig. 4.**
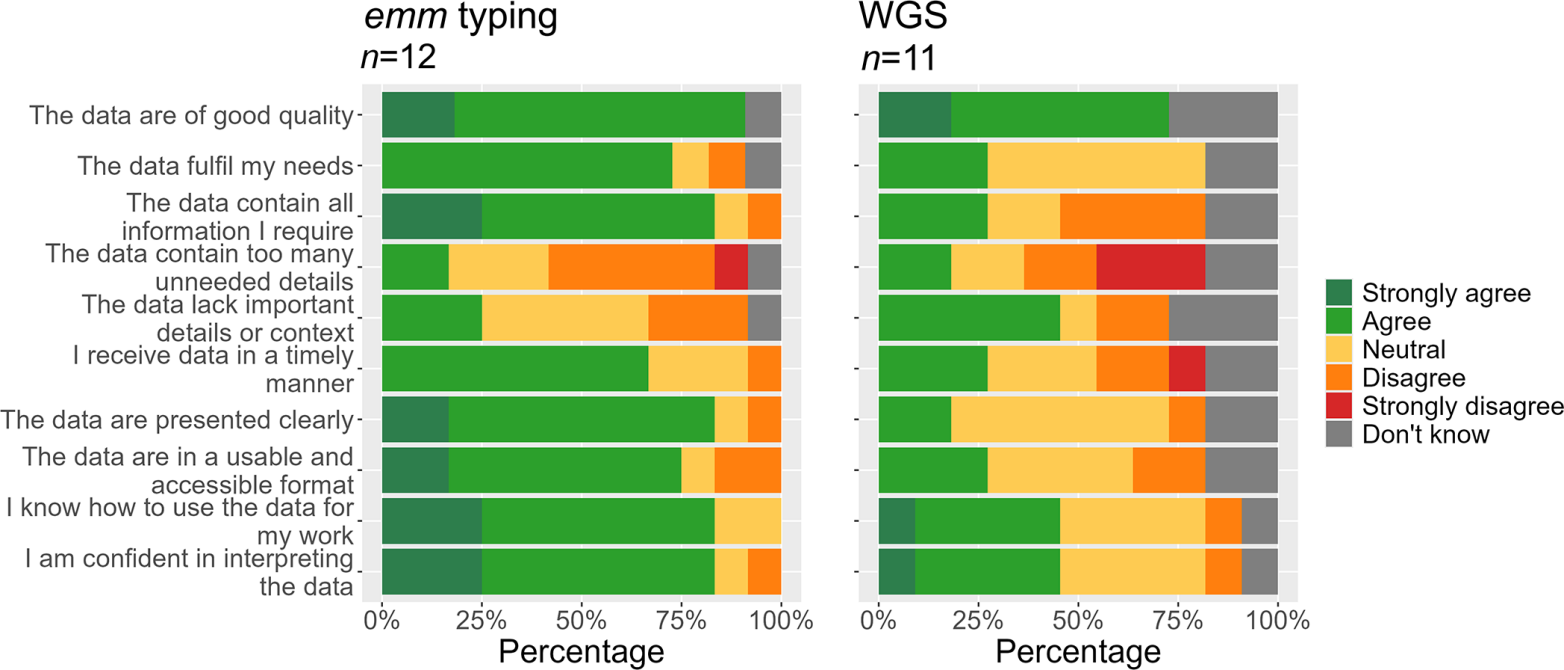
Service user perception of *emm* typing and WGS data timeliness, appropriateness and utility. N denotes the total number of unique respondents. *Emm* typing refers to Sanger sequencing-derived *emm* typing, and WGS refers to SNP-based relatedness analysis. Missing responses were excluded from the analysis. The denominator includes those who answered ‘Don’t know’.

Most survey respondents agreed or strongly agreed that both Sanger *emm* typing (90.9%, *n*=12) and WGS (72.7%, *n*=11) were high quality, but that *emm* typing better met stakeholder current needs (72.7% vs. 27.3%) and was sufficient for health protection activities (83.3% vs. 27.3%). Service users felt that WGS was less timely (27.3% WGS vs. 66.7% for *emm* typing), less clear (18.2% vs. 83.4%), and staff had lower confidence in their WGS data interpretation (45.5% vs. 83.3%). Around 25% of service users selected ‘don’t know’ when asked about WGS, indicating respondents’ limited familiarity with this method (Fig. 4). This may be reflected in the bespoke nature of WGS reporting, with 90% of respondents recommending an improvement in the visualization of SNP data – including the standardization of reporting and the use of online dashboards or direct access to the laboratory information system. Service users also emphasized the need for a better process for requesting bespoke SNP analysis from the reference laboratory, as the current system was seen as ‘resource intense’ and reliant on individuals routinely emailing the laboratory to request analyses and follow up on results.

Most users (91.7%, *n*=12) expressed interest in receiving data on epidemiologically linked cases of the same *emm* type, 75.0% wanted SNP analysis for outbreak-related invasive and non-iGAS cases and 58.3% for all iGAS cases. The provision of SNP data on sporadic, non-outbreak-related cases or on genomic cryptic clusters was less important to users. For cumulative surveillance outputs, a weekly frequency was preferred (83.3%, *n*=6), while WGS SNP data were required as soon as available to support timely outbreak response (75%).

### Implementation of typing data in public health practice

#### Typing data usage for surveillance and outbreak response

Respondents reported using *emm* typing routinely for health protection activities relating to surveillance and outbreak response, including outbreak detection and investigation (both 92.9%, *n*=14), outbreak management (78.6%), exceedance detection (71.4%) and risk assessments (64.3%). Usage was slightly lower for bespoke analysis (50%) and routine regional surveillance reports (42.9%). Fewer respondents (28.6%) used *emm* typing for contact tracing. WGS-derived SNP data were mainly used for outbreak investigation (85.7%) and management (71.4%) and, to a lesser extent, for risk assessment (42.9%). Fewer participants reported usage of SNP data for bespoke analysis (21.4%), cluster detection (21.4%) and contact tracing (14.3%). Where WGS-derived SNP data were used, this was predominantly in addition to *emm* typing. It was not reported to be used for exceedance detection or in routine surveillance reports.

#### Usage of *emm* typing for health protection

Survey participants found *emm* typing most useful in settings with clear epidemiological links, such as care homes, or for rare *emm* types. In community settings, it was often challenging to distinguish linked from sporadic cases when an *emm* type was common.

*We have seen our emm typing based cluster detection tools being too sensitive for common emm types leading to false positive detections of clusters.*

-Service user

Conversely, participants were less confident in using *emm* typing to determine relatedness for common *emm* types without clear epidemiological links:

*It can be difficult to interpret the significance of commonly occurring emm types with confidence. If we are more confident that there is a real link, we may probe epidemiological links more carefully.*

-Service user

When *emm* data supported transmission hypotheses, it strengthened stakeholder engagement and reinforced the need for public health action:

*When cases are the same emm type, this gives more confidence that you need to take action early on and can help external partners realise that they need to participate in the public health response like swabbing and prophylaxis of for example a district nursing team, and that there may be wider IPC [infection prevention and control] issues in a setting/team.*

-Service user

#### Usage of WGS relatedness data for health protection

As SNP analyses had to be requested, WGS SNP data tended to be used in outbreak investigations rather than for routine surveillance. Four survey participants (36.4%, *n*=11) noted that WGS, together with *emm* typing, was more effective than *emm* typing alone in identifying community clusters without clear epidemiological links or within specific populations, such as people who inject drugs.

*WGS can pick up wider community clusters of cases with no apparent epi links. For example, locally by confirming [that] most cases within a wider community emm 59 cluster were linked.*

-Service user

Participants also highlighted the usefulness of WGS SNP data for outbreaks over a wide geographical area or over prolonged timespans. In such cases, WGS provided greater confidence in case relatedness than *emm* typing alone and was used to ‘narrow down’ epidemiologically and/or *emm*-linked cases, helping to target follow-up and public health interventions towards cases with a common infection source.

#### Challenges in using WGS for iGAS surveillance and outbreak management

**Fig. 5.**
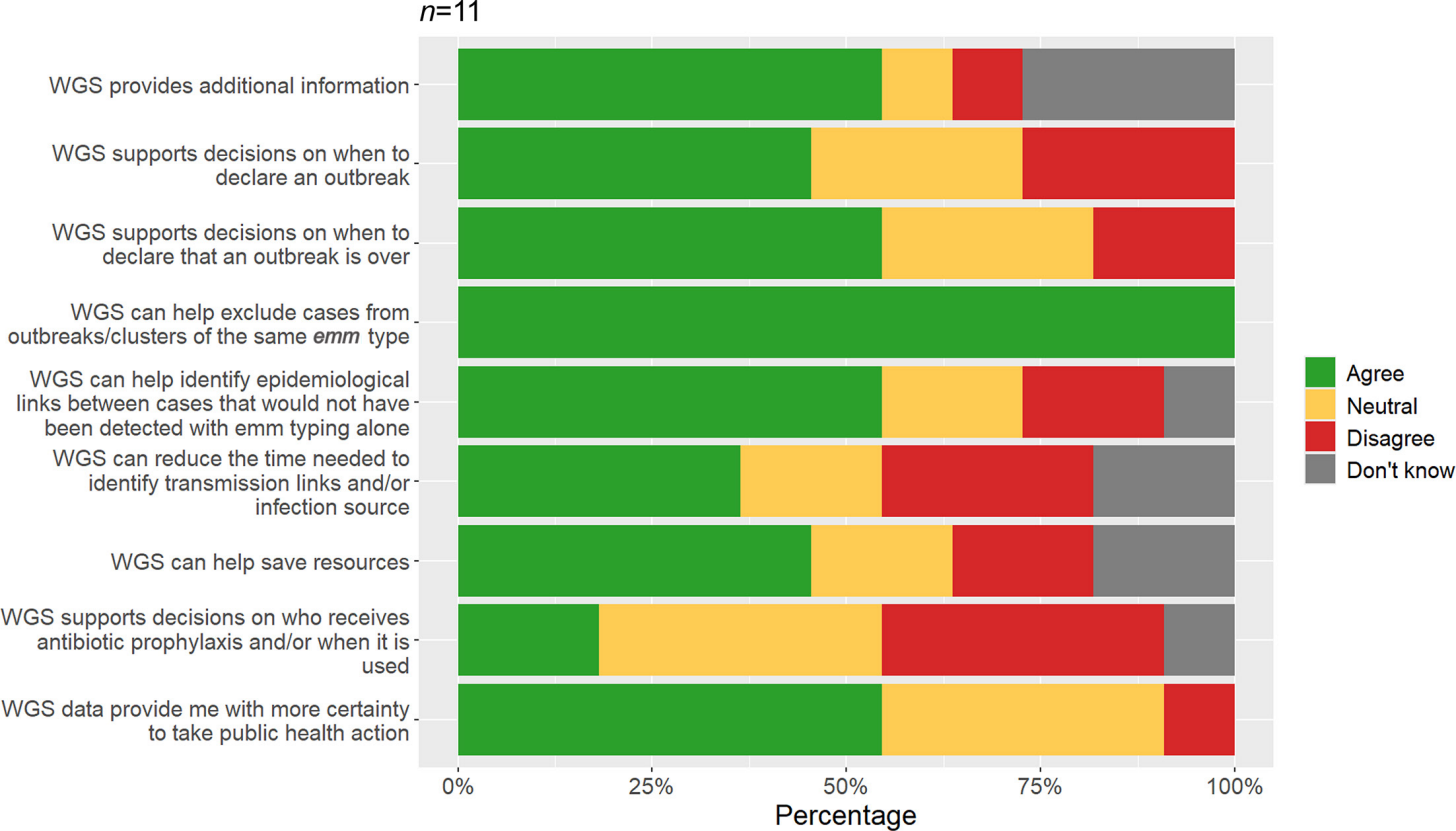
Service users’ agreement with statements regarding the usefulness of WGS for public health practice. N denotes the total number of unique respondents. Missing responses were excluded from the analysis. WGS refers exclusively to WGS-derived SNP analysis. The denominator includes those who answered ‘Don’t know’.

All respondents (*n*=11) agreed that WGS SNP data helped to exclude unrelated cases from outbreaks of the same *emm* type. A majority (54.5%) also felt that WGS could identify links not detectable by *emm* typing alone, providing additional information and greater certainty for public health actions. WGS assisted in decisions on declaring the start (45.5%) or end (54.5%) of outbreaks, and 45.5% agreed that it helps save resources (Fig. 5).

However, longer turnaround times associated with WGS were a concern for service users. Such delays were seen to greatly diminish the usefulness of WGS-derived data to inform immediate public health action, particularly in closed, high-risk settings. Due to its lack of timeliness, WGS was reportedly less useful for reducing the time needed to identify transmission links or infection sources (36.4% agreement, 27.3% disagreement, *n*=11) and in supporting decisions on antibiotic prophylaxis (18.2% agreement, 36.4% disagreement) (Fig. 5).

Nevertheless, respondents did see value in the use of WGS-derived SNP data for community outbreaks where identifying a potential source may be resource-intensive or could be more safely delayed.

*If there was a significant delay in getting WGS data, it would be better to get earlier emm type data on the cases to inform the current risk assessment, than delay decision making on control measures whilst awaiting WGS data. If the decision regarding public health interventions can be safely delayed, it may be worth waiting longer for WGS data […].*

-Service user

The use of SNP-derived WGS data had also led to the identification of genomic clusters without an apparent epidemiological link. Five (41.7%) out of 12 survey participants had previously been informed by the reference laboratory of such cryptic clusters within their region. The identification of cryptic clusters was challenging, given the limited information available, as a genomic link in the absence of epidemiological evidence was not sufficient to inform public health action.

*We had 2 nurseries with almost the same WGS [SNP profiles] – they were about 40 miles apart and we could not identify any epidemiological links. All public health measures had been undertaken already. The WGS result did not add anything to the investigation or management but did make us worry!*

-Service user

Finally, survey participants also highlighted challenges with interpretation of SNP differences greater than 0. SNP data were perceived as complex (41.7%, *n*=12), with one participant mentioning the need for training on data interpretation for proper and efficient usage:

*We need some indication of how many SNPs over what time period is considered to be related as I think this varies by emm type. It would be really useful to have some training or roadshows on the interpretation and use of the results.*

-Service member

### Examination of public health outcomes with implementation of *emm* typing and WGS

**Fig. 6.**
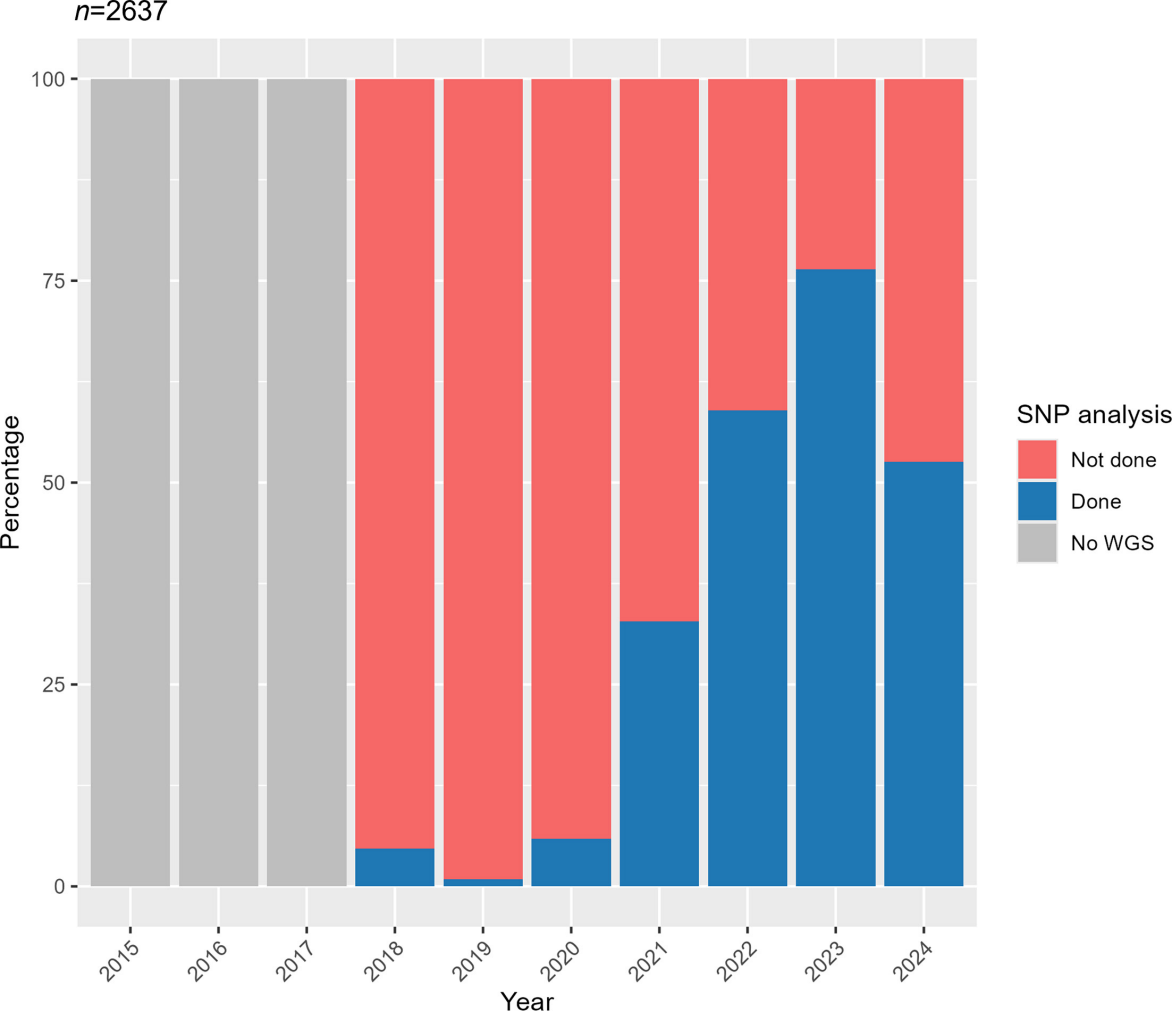
Percentage of outbreak-related isolates for which SNP analysis had been requested. Data for 2024 include only isolates received up to 8 February 2024.

Information of isolates from epidemiologically linked cases (termed outbreak cases) was provided by the reference laboratory (*n*=2,819), of which 2,637 unique isolates were included in the analysis (182 duplicates excluded). WGS sequencing was available for 2,612 (99.1%), of which 1,142 (43.3%) had a SNP analysis available (Fig. 6). All isolates with a SNP analysis also had a Sanger *emm* type recorded. Isolates obtained were from both GAS and iGAS cases (*n*=575, 50.4% iGAS).

The percentage of WGS-sequenced isolates for which a SNP analysis was requested increased annually, from 4.7% in 2018 to 76.4% in 2023. The lower percentage in 2024 (52.6%) is attributed to the lag between isolate reception and the completion of SNP analysis. Additionally, as only isolates up to February were included, this figure does not represent the full year 2024 (Fig. 6).

#### *Emm* and WGS groups within outbreaks

The 1,142 isolates were from 178 outbreaks and clustered into 37 different *emm* types and 371 WGS clusters (isolates within 0–5 SNPs across all outbreaks). Approximately one-third of the 178 outbreaks included isolates of more than one *emm* type (*n*=58; 32.6%), giving 296 distinct epidemiologically linked *emm* clusters consisting of one or more isolates. Of the 371 WGS clusters, 329 (88.7%) spanned only one outbreak, whereas 23 (6.2%) spanned two, 8 (2.2%) spanned three and 11 (3.0%) spanned three or more, indicating cryptic links between outbreaks. Approximately two-thirds of outbreaks (*n*=114; 64.0%) included isolates from more than one WGS cluster, resulting in 491 distinct WGS clusters including one or more isolates where cases had a potential epidemiological link (epidemiologically linked WGS clusters).

#### Genetic diversity within *emm* types

The overall mean number of WGS clusters was 10.0 per *emm* type, with a median of 5 (sd=11.9). *Emm* types more prevalent in the dataset tended to have a higher number of WGS clusters. We also found a wide range of SNP distances within many of the 37 *emm* types identified, highlighting the significant genetic variation present within *emm* types (Table 1). Of the 37 *emm* types, 73.0% exhibited a median SNP difference greater than 5. The genetic distance exceeded five SNPs in 51.1% of clusters, including two or more epidemiologically linked isolates of the same *emm* type (*n*=187). Intra-outbreak SNP distances between isolates of the same *emm* type are available in Appendix 6.

**Table 1.** Overview of SNP distances between outbreak isolates of the same *emm* type Outbreaks show the number of epidemiologically linked clusters per *emm* type, and isolates represent the total number of isolates of the respective *emm* type within these outbreaks. *emm*118 is not displayed as only one sequence with SNP analysis was available.

*Emm* type	Outbreak	Isolate	SNP distance			
			Minimum	Maximum	Mean	Median
*emm*1	32	121	0	7,305	57	39
*emm*33	20	119	0	175	20	12
*emm*89	19	116	0	102	65	69
*emm*12	16	86	0	9,264	540	44
*emm*108	14	81	0	35	13	12
*emm*49	4	61	0	1,005	167	9
*emm*8	6	60	0	26	8	7
*emm*77	9	56	0	5,937	2,535	105
*emm*11	8	51	0	565	229	80
*emm*94	7	40	0	83	50	58
*emm*76	5	39	0	171	27	5
*emm*83	5	35	0	206	55	12
*emm*60	2	31	0	103	27	30
*emm*87	3	29	0	90	41	47
*emm*3	3	28	0	10,261	216	11
*emm*59	1	23	0	9	4	4
*emm*4	4	21	0	8,995	1,088	156
*emm*66	5	16	0	40	27	29
*emm*22	3	15	0	10,537	2,667	81
*emm*53	2	14	0	10	4	4
*emm*75	2	14	0	10,336	3,498	68
*emm*92	2	14	0	20	7	5
*emm*28	1	14	0	111	53	24
*emm*121	2	9	0	33	17	23
*emm*82	2	7	1	9,233	4,782	7,513
*emm*5	1	7	1	242	163	221
*emm*80	1	6	0	4	2	2
*emm*81	1	6	0	8,486	4,810	7,593
*emm*102	1	5	0	10,149	4,060	15
*emm*104	1	5	0	23	17	22
*emm*6	1	3	0	70	46	69
*emm*58	1	3	276	346	310	308
*emm*25	1	2	0	0	0	0
*emm*110	1	2	0	0	0	0
*emm*164	1	2	1	1	1	1

#### Exclusion of outbreak cases using *emm* typing vs. WGS relatedness data

A total of 1,142 isolates encompassed the 178 GAS outbreaks, and *emm* typing was able to exclude 206 isolates (sd=3.5, Interquartile range [IQR]=1) from 58 (32.6%) outbreaks. This included 93 (sd=1.0, IQR=1) sporadic isolates from 50 outbreaks with a different *emm* type to the main *emm* type of the cluster, and 113 isolates (sd=3.0, IQR=0) from 21 outbreaks that formed a secondary *emm* cluster of two or more cases with a different *emm* type to the main *emm* type of the outbreak. WGS SNP analysis was able to exclude 434 isolates (sd=4.2, IQR=3.0) across 114 (68.0%) outbreaks. This included 300 sporadic isolates (sd=2.8, IQR=3) excluded from 107 outbreaks, and an additional 31 outbreaks where 134 isolates (sd=2.3, IQR=0) formed one or more secondary clusters.

Overall, WGS SNP analysis excluded 228 additional isolates than *emm* typing, with a 110.7% increase in total isolates excluded from 96.6% more outbreaks. The greatest benefit was in the exclusion of sporadic cases, with an additional 207 sporadic cases (222.6% increase) across 114.0% more outbreaks identified by WGS, although a small increase in the identification of secondary clusters was also noted (18.6% from 47.6% more outbreaks).

## Discussion

WGS has emerged as a valuable tool for pathogen surveillance and public health response for a range of pathogens. This evaluation found that WGS can enhance iGAS outbreak management through better relatedness resolution; however, its slower turnaround compared with Sanger *emm* typing currently limits its effectiveness and value for rapid health protection responses.

Our results highlight several advantages of WGS over Sanger *emm* typing. Laboratory staff and bioinformaticians favoured a WGS typing approach for its efficiency and the ability to provide more detailed data. Other reference microbiology services within UKHSA already have well-established WGS-based typing services, and a WGS approach is in line with the UKHSA pathogen genomics strategy [24], which aims to integrate genomics into routine public health decision-making and to deliver measurable improvements in outbreak detection, disease control and health security. Additionally, the European Centre for Disease Prevention and Control (ECDC) recommends WGS as the gold standard for microbiological typing of several pathogens due to its enhanced resolution, ease of tracing pathogens across a One Health spectrum and potential for resistance gene surveillance [25,26]. However, Streptococcal species were not prioritized in this guidance [26].

Analysis of WGS SNP data revealed notable genetic diversity within *emm* types, indicating the presence of related populations that could not be distinguished by *emm* typing. Such an enhanced typing resolution allows for more precise microbiological case definitions when investigating outbreaks. According to service users, this was particularly useful when cases were widely dispersed in time or space, spanned various settings or when epidemiological links between the same *emm* type were tenuous.

In this study, WGS provided a more precise definition of outbreaks, as evidenced by a higher exclusion of cases from epidemiologically linked outbreaks compared with *emm* typing. This aligns with existing literature demonstrating WGS-derived relatedness data’s ability to better differentiate true outbreak cases [12,14, 17, 27]. WGS subdivided more outbreaks into two or more smaller clusters than *emm* typing, potentially increasing the workload for public health teams, as these clusters would require separate management. However, by excluding more sporadic cases, WGS can reduce the resources needed for case follow-up. Its enhanced resolution allows for more precise microbiological case definitions when investigating outbreaks and an improved understanding of transmission pathways, supporting more targeted and efficient public health action.

Yet, the longer turnaround times for WGS-based *emm* typing – currently extending from 3 to 5 days for Sanger sequencing to 10–14 days due to differences in their respective technologies – pose significant challenges for timely public health response. Respondents felt that, given the current turnaround times, a WGS-based *emm* typing service would delay public health action and have limited benefits over Sanger typing for GAS outbreak management at the current turnaround speeds. Users also expressed a need for greater standardization and integration of WGS results, including automated reporting through dashboards or direct integration with laboratory systems, to streamline the outbreak detection process and reduce delays.

In terms of surveillance, WGS can be useful for detecting cryptic clusters and tracking resistance genes [9,12, 15, 16]. Although AMR is not yet a major concern for GAS, since clinical penicillin resistance has not been reported, reduced susceptibility to *β*-lactams has been observed in some lineages due to penicillin-binding protein 2x (pbp2x) mutations [28,30]. Some lineages also harbour macrolide resistance genes, with reported prevalence varying by region [31,32]. Resistance to clindamycin, which is important for the treatment of severe GAS infections, has been observed to be increasing and should be closely monitored [32]. However, the potential benefits of WGS for AMR surveillance were not assessed in this study, which focused specifically on outbreak detection and public health response.

Furthermore, among the 371 detected WGS clusters analysed in this study, 11.3% encompassed more than one outbreak, demonstrating WGS’s capability to identify cryptic links, as confirmed by previous research [15,17]. There are documented examples where WGS-based surveillance has improved the detection, investigation and resolution of outbreaks [14,33]. Turner *et al*. [15] found cryptic community transmission and demonstrated WGS’s ability to detect disease clusters in a retrospective UK study [15]. A previous study by Nabarro *et al*. [34], which investigated ten iGAS outbreaks in England, using a subset of isolates that are also examined in the present study [34], highlighted the value of WGS in detecting and managing community healthcare-associated outbreaks, which are often difficult to trace epidemiologically due to cryptic carriage and fomite transmission. They reported that WGS was useful during investigations in excluding unrelated cases of the same *emm* type from epidemiologically linked outbreaks, helping to focus public health actions, particularly for cases with multiple possible GAS exposures. However, their approach combined WGS with Sanger-based *emm* typing [34].

Nevertheless, typing service users emphasized that investigating cryptic clusters detected by WGS was challenging due to a lack of epidemiological information and testing strategies biased towards those with severe illness. Further assessment of the ability of WGS to detect cryptic links and the practical usability of these data is needed.

Overall, this evaluation found that WGS adds value to GAS outbreak response, mainly through its higher typing resolution, detection of cryptic clusters and more comprehensive surveillance capabilities. However, the potential severity of GAS infections demands rapid detection and prompt public health action to stop transmission. Therefore, longer turnaround times diminish much of WGS’s benefit in terms of high typing resolution. Accelerating WGS processes could partially address this issue. For instance, Tagini *et al.* [12] reported that WGS data can be obtained in less than 10 days, and with WGS technology advancement, higher testing speed can be achieved.

However, turnaround times are also strongly influenced by organizational and system-level factors. WGS for GAS at UKHSA is conducted within a central sequencing laboratory that receives isolates of various pathogens from across the agency, resulting in variable throughput and longer routine turnaround times depending on overall demand, competing priorities and seasonal pressures. Outbreak isolates can be prioritized within this system, enabling turnaround times to be reduced temporarily. Optimizing the public health impact of WGS will therefore require not only continued investments in technological and methodological advancements but also sufficient sequencing capacity, prioritization mechanisms and pathways to ensure timely delivery of results during periods of increased demand.

### Limitations

First, the analysis was performed on a subset of all isolates received by the reference laboratory, focusing exclusively on GAS isolates epidemiologically linked to outbreaks and excluding sporadic GAS/iGAS cases. This approach precluded assessing the extent of WGS’s ability to detect cryptic links. While Nabarro *et al.* indicated that WGS can detect cryptic clusters [34], our dataset cannot quantify the added value in this area. While outside of this study’s scope, future analysis including sporadic cases could evaluate how many cryptic clusters can be uncovered through WGS and how this information can be used effectively by outbreak investigators.

Another source of bias arises from the selective application of SNP analysis, which was requested only for certain isolates. Typically, SNP analysis is utilized in prolonged outbreaks with unclear epidemiological links, potentially leading to an overestimation of isolates excluded per outbreak using WGS vs. *emm* typing compared with those outbreaks with clearer links for which SNP analysis was not requested.

Another limitation of our analysis is the use of a fixed five-SNP threshold to define closely related WGS clusters. In real-time investigations, this threshold can vary depending on the context – ranging from as few as two SNPs for small, geographically contained clusters to 10 or even 15 SNPs in other scenarios. As noted above, the genomic diversity of GAS strains may also vary by *emm* type, further complicating the application of a universal SNP threshold.

Moreover, this study relies solely on SNP analysis, the standard method for GAS outbreak investigation at UKHSA. While alternative WGS approaches, such as core genome multilocus sequence typing (cgMLST), which is used by organizations such as the European Food Safety Authority (EFSA) for WGS reporting on food-borne pathogens [35], or k-mer-based methods, may simplify implementation, the relatedness resolution of these methods generally lies above *emm* typing but below SNP analysis. Given the substantial diversity observed among isolates of the same *emm* type in this study, we are confident that a SNP approach is needed to provide the resolution required for outbreak investigation. However, the use of such alternative WGS typing methods could be explored in future work. Furthermore, while survey responses from service users covered all but one region, there were limited free-text responses. This led to a scarcity of detailed examples illustrating how *emm* typing and WGS data specifically impacted public health actions. Despite these limitations, we believe the survey effectively captured the prevailing sentiments and perspectives on Sanger *emm* typing and WGS for GAS outbreak surveillance and response, as evidenced by the consistency of responses across the survey.

This study did not include an economic analysis. Previous systematic reviews have found the evidence on the cost-effectiveness of WGS to be limited, although available studies suggest that WGS can be economically beneficial in surveillance settings when it enables timely intervention [36,37]. However, the applicability of existing economic evaluations to the UKHSA GAS response setting is uncertain, as they were conducted in different contexts (mainly surveillance and clinical settings) and for different pathogens. Future work should therefore incorporate a tailored economic evaluation to provide decision-makers with crucial data regarding the economic implications of implementing WGS over Sanger sequencing for GAS surveillance and outbreak response.

Additionally, this evaluation focused exclusively on the use of WGS for outbreak detection, investigation and informing public health action. Other potential benefits of WGS, such as long-term surveillance monitoring of GAS lineages or tracking AMR, were not assessed or discussed and therefore are not captured in this study.

### Recommendations for public health practice and research

Based on our evaluation, we recommend the following to support effective implementation and use of WGS for iGAS outbreak management and surveillance:

- Maintain some capacity for Sanger *emm* typing in the reference laboratory to enable timely public health actions in outbreak situations when WGS turnaround time is limiting response.
- Automate genomic (i.e. SNP-based) relatedness analysis and deliver it alongside WGS-based *emm* typing to maximize WGS utility.
- Stay aligned with and invest in technological advancements while also testing and evaluating process optimization approaches, alongside ensuring appropriate sequencing capacity in the central laboratory, to achieve meaningful reductions in WGS turnaround times.
- Develop standardized nomenclature with clear guidance on SNP thresholds (overall or for individual *emm* types, informed by individual mutation rates) and their interpretation.
- Further research should aim to better understand genomic diversity within *emm* types and define optimal SNP thresholds for WGS analysis tailored to each type.
- Provide training for service users on interpreting and applying WGS results to support outbreak response and surveillance.
- Involve service users in the co-design of standardized WGS reporting processes to ensure usability and impact of WGS results. Consideration should be given to selecting the most appropriate platform for sharing and visualizing WGS-relatedness results.

## Conclusion

In conclusion, WGS offers significant benefits for GAS surveillance and outbreak response through improved relatedness resolution and detection of cryptic clusters. However, slower turnaround times and data complexity remain key challenges. To maximize impact, we recommend accelerating WGS processes, automating SNP analysis and retaining some Sanger *emm* typing capacity for urgent outbreak situations. Staying up to date with technological advances will be important to further improve turnaround times in the future. Standardized reporting, service user involvement in the design of WGS reporting and targeted training will further support effective public health action. Further research and evaluation are needed to optimize WGS analysis and enhance the use of its results in practice.

## Supplementary material

10.1099/jmm.0.002146 Supplementary Material 1.

## Abbreviations

AMR: antimicrobial resistance
COVID-19: coronavirus disease 2019
ECDC: European Centre for Disease Prevention and Control
EFSA: European Food Safety Authority
FS: field service
GAS: group A *Streptococcus*
HPTs: Health Protection Teams
iGAS: invasive group A *Streptococcus*
PG-PHASE: Pathogen Genomics in Public Health Surveillance Evaluation
SNP: single-nucleotide polymorphism
SSRS: *Staphylococcus* and *Streptococcus* Reference Section
UKHSA: UK Health Security Agency
WGS: whole-genome sequencing
