# Supplementary material for: Evaluating the use of whole-genome sequencing and Sanger emm typing in guiding the identification and public health management of invasive group A Streptococcus clusters and outbreaks in England
Source: J Med Microbiol. 2026 Mar 25;75(3):002146. doi: 10.1099/jmm.0.002146 (PMC13016480; doi:10.1099/jmm.0.002146)
Supplement: Supplementary Material 1. [file jmm-75-02146-s001.pdf]

Appendix 1: PG-PHASE framework

Table S1: Description of the Pathogen Genomics in Public HeAlth Surveillance Evaluation (PG-PHASE) framework for evaluation of WGS implementation in public health practice.

Framework phase	Evaluated aspects
Pre-analysis & analysis	**Laboratory workflows**<br>• Sample section, number of samples processed, costs, turnaround times<br><br>**Impacts of workflow changes**<br>• Impact on staff, capacity, training needs<br>• Appropriateness of changes
Reporting & communication	**Data reporting**<br>• Report timeliness, format, content, frequency, utility<br><br>**Data sharing and communication**<br>• Effectiveness, processes, structures<br>• Feedback from service users to improve practices
Implementation in public health practice	**Data usage by service users**<br>• When, why and how WGS is employed by data users<br>• Ability to utilise and interpret data<br><br>**WGS integration into practice**<br>• Acceptability, usefulness, sustainability of WGS data<br><br>**Public health outcomes**<br>• Case identification, outbreak detection compared to traditional method

Appendix 2: Project map

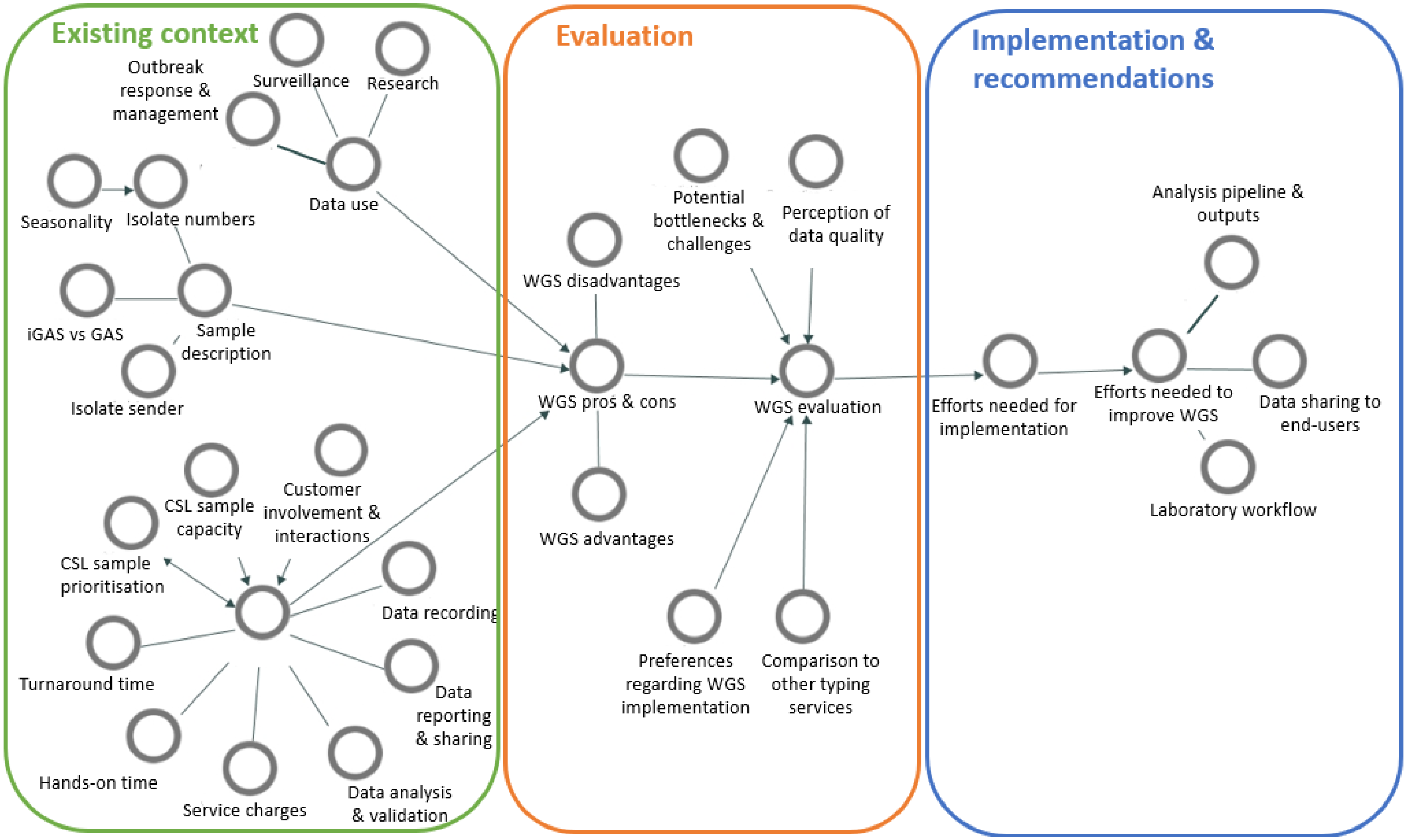

Figure S1: Map of stakeholder interview thematic coding tree.

Coding nodes (grey circles) are organised into three main themes (coloured boxes). Arrow connectors show the influence of one node onto another. Non-directed connectors show associative relationships (for example subcategories of a specific node).

CSL = Central sequencing laboratory, WGS = Whole genome sequencing

Appendix 3: Definition of terms

Table S2: Definition of terms as used in this evaluation

Term	Definition
Case	A person with a laboratory confirmed GAS infection.
Isolate	A biological sample from a GAS case. Multiple isolates can be taken from the same episode and/or the same case.
Outbreak	Isolates which are recorded by SSRS to be epidemiologically linked.
Epidemiologically linked *emm* cluster	Cluster of one or more isolates of the same *emm* type within the same epidemiologically linked outbreak.
WGS cluster	Any isolates within 0-5 SNPs.
Epidemiologically linked WGS clusters	Cluster of one or more isolates within 0-5 SNPs within the same epidemiologically linked outbreak.

Appendix 4: Detailed laboratory processes for iGAS typing

Overview of typing processes & workflows

The UK Health Security Agency (UKHSA) GAS typing service is part of the *Staphylococcus* and *Streptococcus* reference section (SSRS) at UKHSA Colindale, London. NHS Trusts are asked to send all routinely identified GAS isolates from iGAS cases to SSRS for surveillance purposes, with outbreak-related GAS isolates also sent from UKHSA regional laboratories to support outbreak response. The reference laboratory receives between 2,000 and 3,500 GAS isolates from England and Wales each year. The number of samples show seasonal fluctuations with around 80-90 samples per week during the peak of GAS activity between January and March. Sanger sequencing-based emm typing has been the standard service offered for all GAS isolates sent to the laboratory for testing since March 2010. Since September 2022 all received isolates also routinely undergo WGS in addition to emm typing.

Samples sent to the reference laboratory are received at the Central Specimen Reception (CSR) as pure cultures from submitting laboratories. Isolates are plated on the day of reception and cultured overnight before being processed for sequencing the next day.

Current laboratory and analysis processes for Sanger emm typing

For Sanger-based *emm* typing, DNA is extracted manually and an *emm* gene PCR performed to amplify and purify the target DNA region. After PCR, samples are submitted to the Colindale Sequencing Laboratory (CSL) - usually on the same day. CSL purifies the PCR products and then runs the Sanger sequencing, which usually takes 1-3 days. Results are sent from CSL to SSRS where they are analysed by SSRS laboratory staff using *Bionumerics* software. This process takes about 20min after which the detected *emm* type is uploaded into MOLIS, UKHSA's central Laboratory Information Management System (LIMS) for reference microbiology. Technical and medical validation of the result is carried out by senior members of

the reference laboratory before reporting of results to service users (clinicians, epidemiologist, health protection teams). The published turn-around time for Sanger-based *emm* typing is 8 days (1) and Sanger-based emm typing is currently performed daily.

Current laboratory and analysis processes for Whole Genome Sequencing

The WGS workflow also starts with DNA extraction on day two. DNA extraction and purification is automated using QIA*symphony* instruments, with the process taking a full day. WGS samples are therefore usually submitted to the CSL on day three, one day later than for *emm* typing. WGS is currently performed only twice a week due to staffing capacity and prioritisation of Sanger *emm* typing; this can further delay submission to CSL by 1-2 days. CSL performs WGS on the isolates and results are sent directly into a bioinformatic analysis pipeline hosted by Core Bioinformatics (**Figure S2**) which runs in approximately 4 hours.

The analysis pipeline starts with pre-processing and quality assessment of sequencing files. Raw reads are trimmed using Trimmomatic (2), followed by quality assessment using FastQC (3). Trimmed reads are then assessed using KmerID (available here: https://github.com/ukhsa-collaboration/kmerid) to assure the correct species has been sequenced and to check for contamination. Following QC and contamination checks, multi locus sequence typing (MLST) is performed using an internal MLST tool, assembled using Spades (4) , assembly quality assessed using BUSCO (5), before *emm* typing is performed using emmtyper (available here: https://github.com/MDU-PHL/emmtyper).

The *emm* typing result from WGS is exported and manually uploaded into MOLIS and validated by reference laboratory staff if quality standards were met. Full sequencing files are stored in an internal UKHSA server (**Figure S2**).

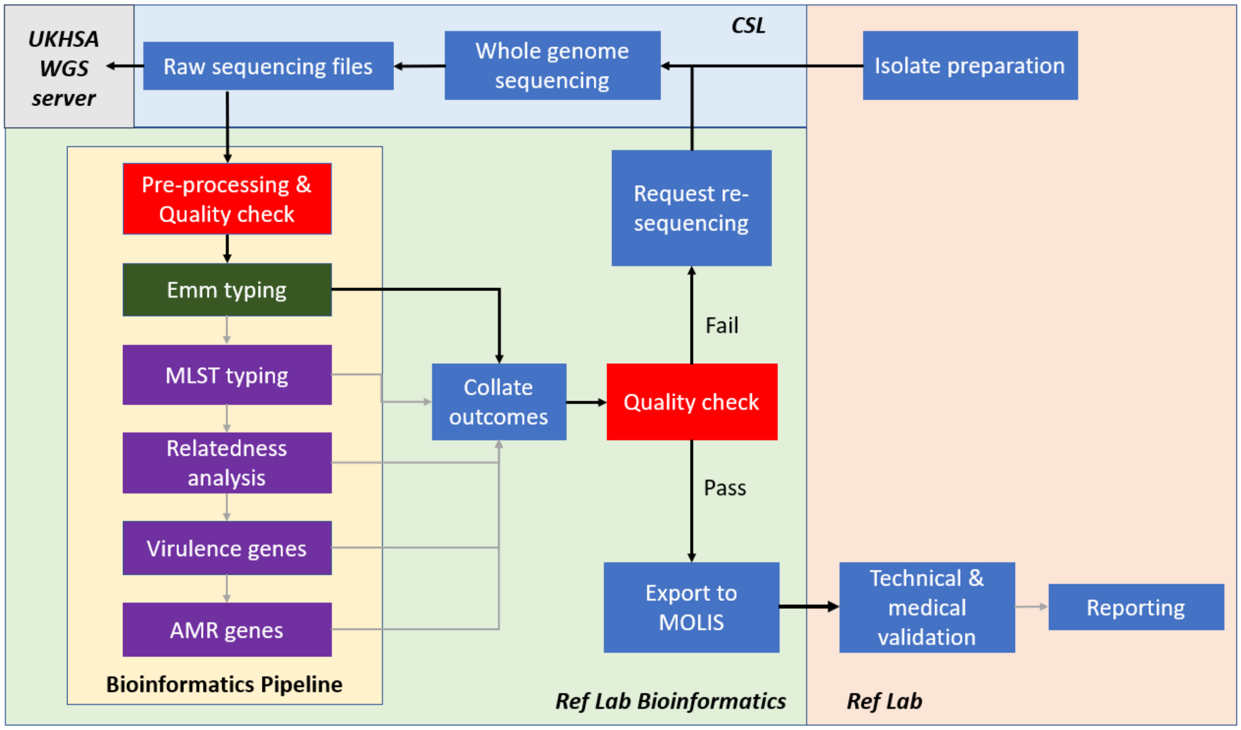

Figure S2: Schematic of bioinformatics pipeline for analysis workflow of whole genome sequencing data.

Black arrows indicate processes which are currently in place. Grey arrows indicate processes still under development and not routinely used. Ref Lab = Reference laboratory, CSL = Colindale Sequencing Laboratory

Currently, the workflow ends at this stage as presently no data derived from WGS typing is reported to UKHSA service users, including *emm* typing which is currently provided by Sanger sequencing only. Multi-locus sequence typing (MLST), an alternative to emm typing using multiple housekeeping genes (5), which is integrated into the pipeline, will be reportable in the future to provide further strain characterisation. Other future developments include reporting of virulence and resistance genes (**Figure S2**). The maximum turnaround time for WGS-based emm typing is 14 days.

Additional WGS analysis can be performed on the sequencing data. This tends to be Single Nucleotide Polymorphism (SNP) analysis, which involves the genomes of requested isolates being compared to generate a matrix of SNP differences from which relatedness can be inferred (**Figure S3**). SNP analysis is performed using the PHEnix pipeline (available at:

https://github.com/ukhsa-collaboration/PHEnix) using BWA (7) for aligning raw reads to an *emm* type specific reference, followed by GATK (8) for variant calling. Gubbins (9) is then used to remove areas of recombination followed by SNP-dist to calculate a SNP distance matrix for analysed isolates (available at: https://github.com/tseemann/snp-dists) and RAXML (10) to create a phylogenetic tree.

Figure S3: Example of an SNP matrix

Both the X and Y axes represent unique isolates with reference indicating the reference strain used. Numbers within the matrix indicate the SNP differences between the samples. The colour scale reflects the relatedness of the isolates, with red indicating close relatedness and blue representing more distant relationships.

Since SNP analysis is not routinely performed, HPTs typically need to request these bespoke analyses via email to SSRS. Only in rare cases, when SSRS identifies unusual activity in a region, does it proactively conduct the analysis to alert HPTs about detected WGS clusters. Conducting SNP analysis can take a few days, depending on bioinformatician capacity and the number of isolates included in the analysis.

Reference laboratory staff tend to use a threshold of 0-3 SNP differences to indicate genetically relatedness in a small number of isolates that occur over a short period of time, and 0-5 SNPs for large clusters occurring over a longer period of time. Outside of this threshold, isolates are not considered to be closely related, and it is therefore unlikely that cases have a common source of infection. However, reference laboratory staff commented that it was very challenging to implement standardised thresholds as mutation rates vary between emm types and SNP thresholds may need to be adjusted for the duration of the cluster, which can persist over an extended period of time.

> *"[The appropriate SNP difference to define a related cluster] is a bit of a moving target really, because it depends on the mutation rate of that sub-strain [i.e. emm type] or also looks at like the length of the outbreak. If it's a particularly protracted one, we might have*

used a cut off of zero to three at the beginning, but it might be more zero to 6 now, just because it's been evolving during that time of studying it.

So yeah, it is a moving target basically. So it's hard to give the exact cut off after which we can ignore it and safely say [two isolates] are unrelated."

-Reference laboratory member

References

1. Agency UHS. Bacteriology Reference Department User Manual. User manual. UKHSA, Division PHM; 2023.

2. Bolger AM, Lohse M, Usadel B. Trimmomatic: a flexible trimmer for Illumina sequence data. Bioinformatics. 2014 Aug 1;30(15):2114-20. doi: 10.1093/bioinformatics/btu170. Epub 2014 Apr 1. PMID: 24695404; PMCID: PMC4103590.

3. Bittencourt SA. *FASTQC : A quality control tool for high throughput sequence data*. 2010

4. Bankevich A, Nurk S, Antipov D, Gurevich AA, Dvorkin M, Kulikov AS, Lesin VM, Nikolenko SI, Pham S, Prjibelski AD, Pyshkin AV, Sirotkin AV, Vyahhi N, Tesler G, Alekseyev MA, Pevzner PA. SPAdes: a new genome assembly algorithm and its applications to single-cell sequencing. J Comput Biol. 2012 May;19(5):455-77. doi: 10.1089/cmb.2012.0021. Epub 2012 Apr 16. PMID: 22506599; PMCID: PMC3342519.

5. Fredrik Tegenfeldt, Dmitry Kuznetsov, Mosè Manni, Matthew Berkeley, Evgeny M Zdobnov, Evgenia V Kriventseva, OrthoDB and BUSCO update: annotation of orthologs with wider sampling of genomes, *Nucleic Acids Research*, 6 January 2025;53(D1):D516–D522, https://doi.org/10.1093/nar/gkae987

6. M.C.J. Maiden, J.A. Bygraves, E. Feil, G. Morelli, J.E. Russell, R. Urwin, Q. Zhang, J. Zhou, K. Zurth, D.A. Caugant, I.M. Feavers, M. Achtman, & B.G. Spratt, Multilocus sequence typing: A portable approach to the identification of clones within populations of pathogenic microorganisms, Proc. Natl. Acad. Sci. U.S.A. 1998;95(6):3140-3145, https://doi.org/10.1073/pnas.95.6.3140 .

7. Li H. Aligning sequence reads, clone sequences and assembly contigs with BWA-MEM. arXiv preprint arXiv:1303.3997. 2013 Mar 16.

8. Van der Auwera, G.A., Carneiro, M.O., Hartl, C., Poplin, R., del Angel, G., Levy-Moonshine, A., Jordan, T., Shakir, K., Roazen, D., Thibault, J., Banks, E., Garimella, K.V., Altshuler, D., Gabriel, S. and DePristo, M.A. 2013, From FastQ Data to High-Confidence Variant Calls: The Genome Analysis Toolkit Best Practices Pipeline. Current Protocols in Bioinformatics, 43: 11.10.1-11.10.33. https://doi.org/10.1002/0471250953.bi1110s43

9. Nicholas J. Croucher, Andrew J. Page, Thomas R. Connor, Aidan J. Delaney, Jacqueline A. Keane, Stephen D. Bentley, Julian Parkhill, Simon R. Harris, Rapid phylogenetic analysis of large samples of recombinant bacterial whole genome sequences using Gubbins, *Nucleic Acids Research*, 18 February 2015;43(3):e15, https://doi.org/10.1093/nar/gku1196

10. Alexey M Kozlov, Diego Darriba, Tomáš Flouri, Benoit Morel, Alexandros Stamatakis, RAxML-NG: a fast, scalable and user-friendly tool for maximum likelihood phylogenetic inference, *Bioinformatics*. November 2019;35(21):4453–4455, https://doi.org/10.1093/bioinformatics/btz305

Appendix 5: Detailed iGAS typing reporting processes

Current reporting processes for Sanger emm typing

Once results for *emm* typing uploaded into MOLIS are technically and medically validated, UKHSA service users are sent a report via eLabs for each analysed isolate. By default, UKHSA service users receive reports for all isolates that meet the iGAS case definition and any isolates linked to outbreaks.

Current reporting processes for WGS

Currently, WGS-derived emm typing results are not routinely provided to UKHSA service users. Bespoke WGS relatedness results are reported back to requesters via email. Additionally, reference laboratory staff regularly attend outbreak management meetings, where they provide expert advice on isolate relatedness.

Changes to data reporting processes upon implementation of WGS as standard service

In the future, if WGS replaces Sanger *emm* typing as the primary service, it is anticipated that emm typing results obtained via WGS will be disseminated to UKHSA service users via eLabs, as is currently implemented for Sanger emm typing. Efforts are being made to expand on the scope of the WGS-derived typing report by including automated SNP-based relatedness information. SSRS are developing a `SNP address`, a strain level nomenclature based on single nucleotide differences between the isolate and a reference strain. The SNP address allows for allocation of isolates confers clear relatedness information without the need to compare isolates to each other (21).

As WGS generates additional typing data over *emm* type it can be used to detect clusters where isolates do not have known epidemiological links, known as ‘cryptic clusters.

“We want to be able to be in a position where we not only respond, so be reactive, but we also want to be able to identify cases where samples are linked […]. And for that to happen, we either have to undertake huge amount [of] analysis with a huge number of samples collected over time, which in the current set-up is very challenging, as there is a maximum number [of samples] that you can actually put in a phylogenetic tree for it to be viable. Or, something that we were trying to develop is to identify or develop a [relatedness] nomenclature so that we don’t necessarily always have to map against a reference. So we're working on that development of a nomenclature, a system for genomic relationship, and then compare samples from various years.”

-Reference laboratory member

There are currently no clear timelines as to when additional data derived from WGS will be routinely shared with UKHSA service users.

Appendix 6: *Intra-outbreak SNP distances between isolates of the same emm type*

Outbreak ID	*emm* type	Sample size	SNP distance			
			Minimum	Maximum	Mean	Median
OB1	emm1	2	2	2	2	2
OB2	emm1	5	0	10	4	1
OB3	emm1	1	N/A	N/A	N/A	N/A
OB4	emm1	2	25	25	25	25
OB5	emm1	3	0	32	21.33	32
OB6	emm1	3	N/A	N/A	N/A	N/A
OB7	emm1	7	0	45	13.1	2
OB8	emm1	1	N/A	N/A	N/A	N/A
OB9	emm1	3	0	0	0	0
OB10	emm1	2	1	1	1	1
OB11	emm1	1	N/A	N/A	N/A	N/A
OB12	emm1	2	0	0	0	0
OB13	emm1	8	0	1	0.25	0
OB14	emm1	2	0	0	0	0
OB15	emm1	2	0	0	0	0
OB16	emm1	3	3	38	26	37
OB17	emm1	5	0	1	0.4	0
OB18	emm1	3	36	92	71.33	86
OB19	emm1	1	N/A	N/A	N/A	N/A
OB20	emm1	1	N/A	N/A	N/A	N/A
OB21	emm1	3	0	6	3.67	5
OB22	emm1	2	39	39	39	39
OB23	emm1	2	0	0	0	0
OB24	emm1	2	30	30	30	30
OB25	emm1	3	34	60	48.67	52
OB26	emm1	2	5	5	5	5
OB27	emm1	1	N/A	N/A	N/A	N/A
OB28	emm1	5	0	42	25	34
OB29	emm1	3	0	2	1.33	2
OB30	emm1	1	N/A	N/A	N/A	N/A
OB31	emm1	1	N/A	N/A	N/A	N/A
OB32	emm1	1	N/A	N/A	N/A	N/A

OB33	emm1	7	0	61	39.71	53
OB34	emm1	1	N/A	N/A	N/A	N/A
OB35	emm1	1	N/A	N/A	N/A	N/A
OB36	emm1	3	0	45	30	45
OB37	emm1	6	0	3	1.2	1
OB38	emm1	1	N/A	N/A	N/A	N/A
OB39	emm1	3	1	35	23.33	34
OB40	emm1	2	0	0	0	0
OB41	emm1	2	43	43	43	43
OB42	emm1	1	N/A	N/A	N/A	N/A
OB43	emm1	2	74	74	74	74
OB44	emm1	2	1	1	1	1
OB45	emm1	3	72	100	83.33	78
OB46	emm1	1	N/A	N/A	N/A	N/A
OB47	emm1	3	0	1	0.67	1
OB48	emm3	20	0	10261	364.14	12
OB49	emm3	2	14	14	14	14
OB50	emm3	5	0	3	0.7	0
OB51	emm3	1	N/A	N/A	N/A	N/A
OB52	emm4	5	0	72	29.1	1.5
OB53	emm4	1	N/A	N/A	N/A	N/A
OB54	emm4	4	0	74	48	53.5
OB55	emm4	2	154	154	154	154
OB56	emm4	1	N/A	N/A	N/A	N/A
OB57	emm4	1	N/A	N/A	N/A	N/A
OB58	emm4	5	0	8988	3671.4	186.5
OB59	emm4	1	N/A	N/A	N/A	N/A
OB60	emm4	1	N/A	N/A	N/A	N/A
OB61	emm5	7	1	242	162.95	221
OB62	emm6	3	0	70	46.33	69
OB63	emm8	8	0	8	3.43	1
OB64	emm8	1	N/A	N/A	N/A	N/A
OB65	emm8	1	N/A	N/A	N/A	N/A
OB66	emm8	4	0	5	2.33	1.5
OB67	emm8	1	N/A	N/A	N/A	N/A
OB68	emm8	1	N/A	N/A	N/A	N/A
OB69	emm8	1	N/A	N/A	N/A	N/A
OB70	emm8	10	0	14	5.84	3
OB71	emm8	13	1	13	5.9	5
OB72	emm8	3	0	0	0	0

OB73	emm8	1	N/A	N/A	N/A	N/A
OB74	emm8	16	0	12	5.71	7
OB75	emm11	10	2	432	137.56	86
OB76	emm11	1	N/A	N/A	N/A	N/A
OB77	emm11	4	0	81	40.5	40.5
OB78	emm11	2	1	1	1	1
OB79	emm11	9	0	126	84.25	95.5
OB80	emm11	3	2	6	4	4
OB81	emm11	2	0	0	0	0
OB82	emm11	2	0	0	0	0
OB83	emm11	18	0	45	9.29	2
OB84	emm12	3	0	52	33.67	49
OB85	emm12	1	N/A	N/A	N/A	N/A
OB86	emm12	4	2	22	12.5	13
OB87	emm12	5	0	19	8.5	3
OB88	emm12	4	1	5	3	3
OB89	emm12	1	N/A	N/A	N/A	N/A
OB90	emm12	11	0	165	30.18	2
OB91	emm12	12	0	142	48.86	15.5
OB92	emm12	2	7	7	7	7
OB93	emm12	1	N/A	N/A	N/A	N/A
OB94	emm12	1	N/A	N/A	N/A	N/A
OB95	emm12	6	0	52	30.87	45
OB96	emm12	2	12	12	12	12
OB97	emm12	5	0	207	97.5	49
OB98	emm12	2	207	207	207	207
OB99	emm12	1	N/A	N/A	N/A	N/A
OB100	emm12	2	131	131	131	131
OB101	emm12	7	1	8	3.9	4
OB102	emm12	2	0	0	0	0
OB103	emm12	2	2	2	2	2
OB104	emm12	1	N/A	N/A	N/A	N/A
OB105	emm12	1	N/A	N/A	N/A	N/A
OB106	emm12	1	N/A	N/A	N/A	N/A
OB107	emm12	6	0	21	11.31	18
OB108	emm12	1	N/A	N/A	N/A	N/A
OB109	emm12	1	N/A	N/A	N/A	N/A
OB110	emm12	1	N/A	N/A	N/A	N/A
OB111	emm22	8	0	189	103.5	68
OB112	emm22	2	1	1	1	1

OB113	emm22	1	N/A	N/A	N/A	N/A
OB114	emm22	1	N/A	N/A	N/A	N/A
OB115	emm22	1	N/A	N/A	N/A	N/A
OB116	emm22	2	0	0	0	0
OB117	emm25	2	0	0	0	0
OB118	emm28	10	0	6	2.93	3
OB119	emm28	1	N/A	N/A	N/A	N/A
OB120	emm28	1	N/A	N/A	N/A	N/A
OB121	emm28	1	N/A	N/A	N/A	N/A
OB122	emm28	1	N/A	N/A	N/A	N/A
OB123	emm33	1	N/A	N/A	N/A	N/A
OB124	emm33	9	1	170	72.22	25.5
OB125	emm33	2	0	0	0	0
OB126	emm33	12	0	169	29.82	3
OB127	emm33	2	9	9	9	9
OB128	emm33	1	N/A	N/A	N/A	N/A
OB129	emm33	1	N/A	N/A	N/A	N/A
OB130	emm33	5	1	5	3.2	3.5
OB131	emm33	2	14	14	14	14
OB132	emm33	15	0	22	11.26	13
OB133	emm33	2	3	3	3	3
OB134	emm33	2	1	1	1	1
OB135	emm33	1	N/A	N/A	N/A	N/A
OB136	emm33	5	0	1	0.4	0
OB137	emm33	8	0	4	1.79	2
OB138	emm33	2	12	12	12	12
OB139	emm33	2	30	30	30	30
OB140	emm33	7	0	6	2.67	2
OB141	emm33	1	N/A	N/A	N/A	N/A
OB142	emm33	4	1	4	2.5	2.5
OB143	emm33	3	0	0	0	0
OB144	emm33	1	N/A	N/A	N/A	N/A
OB145	emm33	1	N/A	N/A	N/A	N/A
OB146	emm33	2	9	9	9	9
OB147	emm33	2	2	2	2	2
OB148	emm33	20	0	2	0.76	1
OB149	emm33	6	0	15	7.47	13
OB150	emm49	2	3	3	3	3
OB151	emm49	5	1	12	7.8	10.5
OB152	emm49	6	4	908	331.73	61

OB153	emm49	48	0	41	8.34	8
OB154	emm53	1	N/A	N/A	N/A	N/A
OB155	emm53	2	0	0	0	0
OB156	emm53	11	0	8	3.4	3
OB157	emm58	1	N/A	N/A	N/A	N/A
OB158	emm58	2	308	308	308	308
OB159	emm59	1	N/A	N/A	N/A	N/A
OB160	emm59	22	0	9	3.96	4
OB161	emm60	16	0	84	13.65	5
OB162	emm60	1	N/A	N/A	N/A	N/A
OB163	emm60	14	0	5	1.24	1
OB164	emm66	2	10	10	10	10
OB165	emm66	1	N/A	N/A	N/A	N/A
OB166	emm66	3	0	32	21	31
OB167	emm66	2	0	0	0	0
OB168	emm66	1	N/A	N/A	N/A	N/A
OB169	emm66	2	0	0	0	0
OB170	emm66	4	3	14	8.5	9
OB171	emm66	1	N/A	N/A	N/A	N/A
OB172	emm75	1	N/A	N/A	N/A	N/A
OB173	emm75	1	N/A	N/A	N/A	N/A
OB174	emm75	1	N/A	N/A	N/A	N/A
OB175	emm75	1	N/A	N/A	N/A	N/A
OB176	emm75	1	N/A	N/A	N/A	N/A
OB177	emm75	4	1	45	22.67	22.5
OB178	emm75	1	N/A	N/A	N/A	N/A
OB179	emm75	3	0	0	0	0
OB180	emm75	1	N/A	N/A	N/A	N/A
OB181	emm76	11	0	10	4.51	4
OB182	emm76	2	99	99	99	99
OB183	emm76	9	0	140	33.61	5
OB184	emm76	11	0	164	32.33	3
OB185	emm76	6	0	7	3.4	4
OB186	emm77	2	0	0	0	0
OB187	emm77	2	2	2	2	2
OB188	emm77	1	N/A	N/A	N/A	N/A
OB189	emm77	27	0	5937	1431.28	6
OB190	emm77	1	N/A	N/A	N/A	N/A
OB191	emm77	2	16	16	16	16
OB192	emm77	2	3	3	3	3

OB193	emm77	2	16	16	16	16
OB194	emm77	2	5718	5718	5718	5718
OB195	emm77	1	N/A	N/A	N/A	N/A
OB196	emm77	7	0	2	0.86	1
OB197	emm77	1	N/A	N/A	N/A	N/A
OB198	emm77	1	N/A	N/A	N/A	N/A
OB199	emm77	1	N/A	N/A	N/A	N/A
OB200	emm77	3	3	5160	3440	5157
OB201	emm77	1	N/A	N/A	N/A	N/A
OB202	emm80	6	0	4	1.93	2
OB203	emm81	1	N/A	N/A	N/A	N/A
OB204	emm81	2	7874	7874	7874	7874
OB205	emm81	1	N/A	N/A	N/A	N/A
OB206	emm81	1	N/A	N/A	N/A	N/A
OB207	emm81	1	N/A	N/A	N/A	N/A
OB208	emm82	4	1	8340	3915.5	3758.5
OB209	emm82	2	39	39	39	39
OB210	emm82	1	N/A	N/A	N/A	N/A
OB211	emm83	2	0	0	0	0
OB212	emm83	1	N/A	N/A	N/A	N/A
OB213	emm83	1	N/A	N/A	N/A	N/A
OB214	emm83	7	3	13	9.71	9
OB215	emm83	4	6	16	11	11
OB216	emm83	15	0	11	6.1	7
OB217	emm83	1	N/A	N/A	N/A	N/A
OB218	emm83	1	N/A	N/A	N/A	N/A
OB219	emm83	1	N/A	N/A	N/A	N/A
OB220	emm83	2	10	10	10	10
OB221	emm87	5	0	3	1.6	1.5
OB222	emm87	1	N/A	N/A	N/A	N/A
OB223	emm87	4	5	9	6.33	5.5
OB224	emm87	1	N/A	N/A	N/A	N/A
OB225	emm87	1	N/A	N/A	N/A	N/A
OB226	emm87	1	N/A	N/A	N/A	N/A
OB227	emm87	13	0	32	5.38	1
OB228	emm87	1	N/A	N/A	N/A	N/A
OB229	emm87	1	N/A	N/A	N/A	N/A
OB230	emm87	1	N/A	N/A	N/A	N/A
OB231	emm89	2	1	1	1	1
OB232	emm89	3	27	41	35.33	38

OB233	emm89	3	5	51	34	46
OB234	emm89	2	1	1	1	1
OB235	emm89	2	55	55	55	55
OB236	emm89	15	0	82	40.05	62
OB237	emm89	7	0	83	28.48	16
OB238	emm89	1	N/A	N/A	N/A	N/A
OB239	emm89	1	N/A	N/A	N/A	N/A
OB240	emm89	3	64	72	68.67	70
OB241	emm89	2	0	0	0	0
OB242	emm89	10	2	89	57.84	54
OB243	emm89	2	2	2	2	2
OB244	emm89	23	0	97	20.31	5
OB245	emm89	1	N/A	N/A	N/A	N/A
OB246	emm89	1	N/A	N/A	N/A	N/A
OB247	emm89	2	42	42	42	42
OB248	emm89	1	N/A	N/A	N/A	N/A
OB249	emm89	4	47	57	52.2	52
OB250	emm89	8	0	59	17.14	5
OB251	emm89	1	N/A	N/A	N/A	N/A
OB252	emm89	3	0	0	0	0
OB253	emm89	3	0	1	0.67	1
OB254	emm89	1	N/A	N/A	N/A	N/A
OB255	emm89	8	0	85	46.26	49
OB256	emm89	7	1	87	42.48	71
OB257	emm92	12	0	13	3.71	3
OB258	emm92	2	1	1	1	1
OB259	emm94	1	N/A	N/A	N/A	N/A
OB260	emm94	2	37	37	37	37
OB261	emm94	5	0	16	8.8	12
OB262	emm94	14	0	2	0.43	0
OB263	emm94	4	0	62	31	31
OB264	emm94	8	0	13	4.82	3.5
OB265	emm94	2	2	2	2	2
OB266	emm94	4	3	10	6.17	5.5
OB267	emm102	4	0	10149	5073.67	5072
OB268	emm102	1	N/A	N/A	N/A	N/A
OB269	emm104	5	0	23	17.1	22
OB270	emm108	1	N/A	N/A	N/A	N/A
OB271	emm108	3	1	18	11.33	15
OB272	emm108	1	N/A	N/A	N/A	N/A

OB273	emm108	1	N/A	N/A	N/A	N/A
OB274	emm108	4	0	9	6.83	8
OB275	emm108	4	0	12	6	6
OB276	emm108	17	0	13	5.8	7
OB277	emm108	1	N/A	N/A	N/A	N/A
OB278	emm108	2	1	1	1	1
OB279	emm108	11	0	6	3.05	3
OB280	emm108	6	0	18	9.6	15
OB281	emm108	3	1	9	6	8
OB282	emm108	11	0	17	5.13	4
OB283	emm108	2	10	10	10	10
OB284	emm108	4	1	3	1.67	1.5
OB285	emm108	2	0	0	0	0
OB286	emm108	1	N/A	N/A	N/A	N/A
OB287	emm108	5	0	3	1.3	1
OB288	emm108	2	21	21	21	21
OB289	emm110	2	0	0	0	0
OB290	emm118	1	N/A	N/A	N/A	N/A
OB291	emm121	3	16	17	16.67	17
OB292	emm121	6	0	3	1.6	2
OB293	emm164	2	1	1	1	1

Appendix 7: Interview schedule

The following is the interview schedule targeted towards laboratory staff. Interview schedules for SSRS leadership, bioinformaticians and CSL members included the same topics with slight adaptations on the questions depending on roles. These are available on request.

Questions are shown in **bold**, probes which might have been asked during the interview are shown in *italics*.

A. About you

1. **Could you please introduce yourself and state which team you work in?**

B. Job and sample description

2. **What is your job role and what does this role involve?**
    a. **What kind of samples/pathogens do you work with?**
    b. **What % of your time is spent on GAS (if other pathogens mentioned)**

3. **Could you please describe the type of samples you process for emm typing of GAS?**
    a. **Where are they sent from?**
    b. **How often do you receive samples?**

c. **Can you estimate roughly what percentage of isolates are linked to ongoing outbreaks and how many are sporadic cases?**

d. **What percentage of isolates from suspected iGAS cases do you receive?**

e. **Do you receive isolates from non-invasive GAS infections? How many? What would be the reasons for you receiving these?**

4. **Do the samples you process for WGS differ from the samples you process for emm typing?** *(regarding everything mentioned above)*

a. **If yes, then who decides which samples are sent/accepted for WGS? How is this decided?**

5. **How many iGAS/GAS samples do you process per week on average? Does this change over the year? How many are emm typed (%) and how many are sequenced (%)?**

Winter vs summer

6. **How long does it take on average from the time you receive a sample…**

a. **Until you have the results of the laboratory analysis?**

Please estimate for emm typing and WGS separately.

Raw data, before analysis, to be shared with bioinformaticians

C. Workflows

7. Can you give a broad overview of the workflow for emm typing

Probe for:

- *Timing (how long does it take?)*
- *Hands-on time (how many people and who is involved?)*
- *Data interpretation/preparation for reporting/sharing (Who is in charge of that?)*
- *Data sharing/reporting (What is shared, by whom, to whom?)*

8. Can you also give a broad overview of the workflow for WGS

Probe for:

- *Timing (how long does it take?)*
- *Hands-on time (how many people and who is involved?)*
- *Data interpretation/preparation for reporting/sharing (Who is in charge of that?)*
- *Data sharing/reporting (What is shared, by whom, to whom?)*

9. Comparing the two workflows described, what are the main differences for your role and how do you perceive them?

Probe for:

- *Positives/negatives*
- *More work/less work (hands-on time, waiting times, automatizations)*

- *More complicated/less complicated*

D. <u>Sharing and reporting of results</u>

10. Do you record emm typing test results for internal use?

  a. If yes, what is recorded, where and how?

11. Do you send emm typing test results for further analysis or share it with anyone?

  a. If yes, who do you send it to/share it with?

  b. In what form do you send/share them?

    - *Raw data files vs reports*
    - *All data vs partial data*
    - *Anonymised/de-identified?*

12. Do you record WGS test results for internal use? What is recorded?

  a. If yes, what is recorded, where and how?

13. Do you send WGS test results for further analysis or do you share it with anyone else?

  a. If yes, who do you send it to?

  b. In what form do you send them?

- *Raw data files vs reports*
- *All data vs partial data*
- *Anonymised/de-identified?*

E. Impact and barriers to WGS implementation

14. Do you feel that you have all the necessary resources, training and/or equipment to produce high quality WGS analyses?

Probe for:

- *Training needs*
- *Equipment*
- *Guidelines (for analysis, but also interpretation/sharing/documenting of data)*
- Time

a. If not, what is missing?

And how could it be (better) provided?

15. In your opinion, what are the main barriers you see to implementing WGS for all iGAS samples? Why?

Probe for:

- *Time*
- *Staff*

- *Costs*
- *Logistics/priorities/not receiving samples*
- *Data interpretation/sharing/reporting à compatibility with systems for surveillance/case management?*

16. How could these barriers be avoided/handled? What changes/adaptions would you want to see?

17. How confident are you in the quality and accuracy of the data you produce for emm typing? Why?

18. How confident are you in the quality and accuracy of the data you produce for WGS? Why?

How does it compare to the quality of emm typing data?

19. What do you think will be most impacted in your own or your groups work by a complete switch from emm typing to WGS? What will change and how?

- *Daily procedures*
- *Time management/requirements (hands-on time vs automatization)*
- *Reporting/sharing data*
- *Recording data procedures*

20. **Overall, do you think a switch from emm typing to WGS will be positive? Why?**

For you/your work? Your group's work? The end-users of the data?

21. Would you recommend a complete switch from emm typing to WGS or using WGS in conjunction with emm typing? Why?

22. Is there anything else you would like to mention?

Appendix 8: UKHSA service user survey form

This following online survey was distributed to all HPT and FS iGAS leads in England through emailing lists.

Participant information

Background and aims of evaluation

UKHSA's Evaluation and Epidemiological Science (EES) Division are working with colleagues in Clinical and Public Health and Field Services, Health Protection Operations on an evaluation to understand how *emm* typing and whole genome sequencing (WGS) of invasive and non-invasive group A streptococcal (iGAS/GAS) isolates are used to support public health surveillance and outbreak management.

UKHSA iGAS WGS Evaluation Survey

This evaluation aims to understand:

- What typing data is received by Health Protection and Field Service teams in relation to iGAS/GAS
- How this typing data is used
- Whether this typing data could be improved to support public health surveillance and incident management.

Please complete the questionnaire even if you do not currently receive typing data on iGAS/GAS, as it is important that we capture gaps in data provision.

The survey will take approx. 20-30 minutes to complete.

If you are unable to complete the survey in a single sitting, your responses can be saved, and you will return to the first uncompleted page of the survey when you click on the survey link again. Please make sure to hit NEXT to save a completed page before exiting the survey to

avoid having to complete the same page again. You will not be able to amend your answers once the survey has been submitted.

Answers provided will be kept in strict confidence and will be held and processed securely in line with the Data Protection Act 2018 and UKHSA information governance policies and procedures. Reporting of the findings will be anonymised so that individual responses cannot be identified.

If you have any queries about this survey or the evaluation, please contact the UKHSA's Evaluation and Epidemiological Science team via email: [email removed], quoting the reference 'iGAS WGS Evaluation' in the email subject.

<u>Section 1: About you</u>

1. Which team do you work in?

- Field service
- Health protection team   Other, please specify

2. Which UKHSA region(s) do you work in?* Select all that apply

- National
- East of England East Midlands
- London
- North East North West South East South West
- West Midlands Yorkshire and Humber

<u>Section 2: emm typing and WGS data provision</u>

In the following section we will ask you about the use of typing information received from the national reference laboratory for isolates of invasive or non-invasive group A streptococcus (iGAS/GAS). The two data types we are interested in are emm typing and Whole Genome Sequencing (WGS).

3. What kind of typing data do you receive or have access to relating to iGAS/GAS emm typing or WGS?* Please select all that apply and at least one option per column

	**Emm typing**	**WGS**
iGAS case level report from reference lab (reports generated by reference lab for each isolate or case)		
iGAS case level report from HP Zone (typing information included in or attached to HP Zone records)		
GAS case level report from reference lab (reports generated by reference lab for each isolate or case)		
GAS case level report from HPZone (typing information		

included in or attached to HPZone records)		
iGAS/GAS outbreak reports or spreadsheets provided by the reference lab		
iGAS/GAS surveillance for example national iGAS dashboard		
None of the above		

4. Are there any other iGAS/GAS typing data you receive or have access to?

If yes, please specify them below for emm typing and WGS data separately

5. When do you usually receive iGAS/GAS emm typing and/or WGS data?

Please select all that apply

	Emm typing	WGS
Routine reports on individual cases		
Reports when clusters are detected by WGS (cryptic clusters)		

On request		
Ad hoc only (for example during an outbreak)		
Never receive data		

6. Are there other times you receive iGAS/GAS typing data?

If yes, please specify them below for emm typing and WGS separately

7. How often do you receive regular reports on emm typing of iGAS/GAS isolates?

- Daily (Monday - Friday) Weekly
- Monthly Quarterly Yearly
- Other, please specify

8. How often do you receive regular reports on WGS of iGAS/GAS isolates?

- Daily (Monday - Friday) Weekly
- Monthly Quarterly Yearly
- Other, please specify

9. In your region, are emm typing and/or WGS data for GAS/iGAS recorded in a systematic way? Please select all that apply

	Emm typing	WGS
Yes, recorded in case record on HPZone		
Yes, automatic recording from SGSS to national iGAS dashboard		
Yes, automatic recording from SGSS to local dashboards		
Yes, recorded in an iGAS surveillance tracker or other surveillance tool		
No, these data are not recorded systematically		
Don't know		

10. If emm typing or WGS data is recorded in other ways in your region, please specify here:

Please specify for emm typing and WGS data separately

Section 3: Data use for surveillance and public health response

11. Select which type of data you use for the following purposes:* Please select all that apply and at least one per column

	Emm typing	WGS
Contact tracing		
Risk assessment		
Surveillance including exceedance detection		
Cluster/outbreak detection		
Cluster/outbreak investigation		
Cluster/outbreak management		
Routine regional surveillance reports		
Bespoke analysis (e.g. in response to data requests or research projects)		
None of the above		

12. Are there other purposes for which you use emm typing or WGS data?

If yes, please specify for emm typing and WGS data separately

13. Which data type (emm typing or WGS) do you think is most appropriate for the following purposes?

	**Emm typing**	**WGS**	**No preference**	**Neither required**	**Don't know/Not used**
Supporting surveillance activities					
For cluster identification					
Outbreak detection and/or investigation					
Outbreak management					
Informing public health action/ iGAS/GAS response					

<u>Section 4: Use of emm typing data for surveillance activities and exceedance detection</u>

14. How does emm typing data help you undertake surveillance of iGAS/GAS?

- Allows for monitoring of emm trends
- Allows for the detection of potential clusters
- Allows for the identification of cases with the same emm type which are unlinked to the ongoing outbreak
- Allows for the identification of cases which have a different emm type and are unlinked to the ongoing outbreak
- Other, please specify

15. How does emm typing data help in detection of iGAS/GAS clusters or outbreaks?

16. How does information available through WGS in addition to emm typing contribute to iGAS/GAS surveillance?

- Allows for monitoring of phylogenetic trends
- Allows for the detection of potential clusters
- Allows to spot cases with the same emm type which are unlinked to the ongoing outbreak
- Allows to spot cases which have a different emm type and are unlinked to the ongoing outbreak   Other, please specify

17. How does the addition of WGS to emm typing help in detecting iGAS/GAS clusters or outbreaks?

Section 5: Use of WGS data for surveillance activities and exceedance detection

18. In your experience, has the availability and use of WGS in addition to emm typing changed the type of setting or population where clusters/outbreaks are detected?

- Yes
- No
- Don't know

19. If yes, please provide details.

Section 6: Potential use of WGS data for surveillance activities and exceedance

20. How do you think the addition of WGS to emm typing information would help in detecting iGAS/GAS clusters or outbreaks?

21. How do you think the addition of WGS to emm typing information would help in iGAS/GAS surveillance?

Section 7: Use of emm typing data for cluster/outbreak investigation and management

22. How does emm typing data (without WGS) help you investigate and/or manage an iGAS/GAS outbreak?

Section 8: Use of WGS data for cluster/outbreak investigation and management

23. In your experience, has the availability and use of WGS data in addition to emm typing changed the number of clusters/outbreaks detected and/or managed?

- Yes, more clusters/outbreaks
- Yes, fewer clusters/outbreaks
- No change
- Unsure/Don't know

24. In your experience, has the availability and use of WGS in addition to emm typing changed the type or nature of clusters/outbreaks you managed?

Please select all that apply

- Yes clusters/outbreaks are bigger
- Yes, clusters/outbreaks are smaller
- Yes, clusters/outbreaks span more time (i.e., cases are farther away from each other in time)

- Yes, clusters/outbreaks are more geographically dispersed (i.e., cases are geographically further from each other)
- Yes, clusters are detected in multiple settings or populations (i.e., care home and PWID)
- No change
- Unsure/Don't know   Other, please specify

25. In your experience, has the addition of WGS improved the ability to identify whether cases are sporadic (unlinked) or part of a cluster/outbreak when cases of a common emm type are identified?

Please assess this in the following scenarios and select all that apply:

- Yes, for cases where an epidemiological link isn't apparent
- Yes, when cases have a potential epidemiological link
- Yes, in identified clusters or outbreaks where cases have the same emm type in the community
- Yes, in identified clusters or outbreaks where cases have the same emm type in closed settings such as care homes or schools
- No, no improvement

26. Does the addition of WGS data to emm typing data contribute in any other ways to investigating and/or managing iGAS/GAS clusters/outbreaks beyond the ability to identify whether cases are sporadic or part of an outbreak?

27. In your experience, has WGS in addition to emm typing been beneficial in supporting the management of outbreaks in any specific setting and/or population?

- Yes
- No
- Don't know

28. If yes, which setting(s) and/or population(s) in particular?

Please select all that apply

- Care homes
- Prisons
- Schools
- Hospitals
- District nursing
- People who inject drugs
- Other, please specify

29. Please describe how WGS has been beneficial in supporting outbreak management in such setting(s)/population(s)n.

30. Has your team dealt with cryptic clusters (clusters with genetic links detected via WGS, but without epidemiological links)?

- Yes
- No
- Don't know

31. If yes, please describe what you did when a cryptic cluster was detected.

Please describe what you did when a cryptic cluster was detected

Section 9: Use of WGS data for public health action

32. Can you describe how the use of WGS in addition to emm typing has influenced public health action?

Section 10: Potential use of WGS data for cluster/outbreak investigation and management

33. How do you think the addition of WGS data could contribute to investigating and/or managing iGAS/GAS clusters/outbreaks beyond the use of emm typing alone?

Section 11: Comparing the use of emm typing and WGS data for cluster/outbreak investigation and management

34. Please indicate your level of agreement to the following statements about WGS in comparison to emm typing data

	Strongly agree	Agree	Neutral	Disagree	Strongly disagree	Don’t know
WGS data provides me with more certainty to take public health action						
WGS can help exclude cases from outbreaks/clusters of the same emm type						
WGS can help identify epidemiological links between cases that would not have						

been detected with emm typing alone						
WGS can help save resources (swabs, staff time, money) due to reduced need for follow up when cases and their contacts can be excluded from clusters						
WGS supports decisions on who receives antibiotic prophylaxis and/or when it is used						
WGS can reduce the time needed to identify						

transmission links and/or infection source						
WGS supports decisions on when to declare an outbreak						
WGS supports decisions on when to declare that an outbreak is over						
WGS provides additional information (such as on antibiotic resistance) which could potentially be useful to guide decisions on treatment and prophylaxis						

Section 12: Acceptability, appropriateness, timeliness and utility of emm typing data

35. To what extent do you agree or disagree with the following statements about emm typing data for iGAS/GAS you receive in your work?

	**Strongly agree**	**Agree**	**Neutral**	**Disagree**	**Strongly disagree**	**Don't know**
The data received is in a usable and/or accessible format						
The data received is presented clearly						
The data received contains all the appropriate and relevant information I require						
The data received contains too many details I do not need						
The data received lacks key details or context that I require						

The data is received in a timely manner to support public health action						
The data received fulfills my needs						
The data received is of good quality						
I am confident at interpreting the data reports I receive						
I understand what the reported results mean and how I can use them to guide decisions in my work						

Section 13: Acceptability, appropriateness, timeliness and utility of WGS data

36. To what extent do you agree and disagree with the following statements about WGS data for iGAS/GAS?

	**Strongly agree**	**Agree**	**Neutral**	**Disagree**	**Strongly disagree**	**Don’t know**
The data received is in a usable and/or accessible format						
The data received is presented clearly						
The data received contains all the appropriate and relevant information I require						
The data received contains too many details I do not need						
The data received lacks key details or context that I require						
The data is received in a timely manner to support public health action						
The data received fulfills my needs						

The data received is of good quality						
I am confident at interpreting the data reports I receive						
I understand what the reported results mean and how I can use them to guide decisions in my work						

Section 14: Improving acceptability, appropriateness, timeliness and utility of WGS data

37. For which iGAS or GAS cases is or would it be useful for you to receive WGS data of isolates in addition to emm typing results?

Please select all that apply

- For all detected iGAS cases (sporadic & outbreak)
- For all detected GAS cases (sporadic & outbreak)
- New detected iGAS case associated with ongoing outbreak
- New detected GAS case associated with ongoing outbreak
- For iGAS/GAS cases with the same emm type, epidemiologically linked

- For iGAS/GAS cases with the same emm type, not epidemiologically linked
- For any sporadic iGAS/GAS cases
- Other, please specify

38. When would you like to receive iGAS/GAS emm typing and/or WGS data?

Please select all that apply

- Ad hoc (during an outbreak)
- Regular reports on all typed/sequenced isolates in a specific time frame
- Sporadic reports on individual cases
- Sporadic reports when clusters are detected by WGS   On request
- Other, please specify

39. If you would like regular reports, how often would you want to receive them?

- Daily
- Weekly
- Monthly
- Quarterly
- Yearly
- Other, please specify

40. How could the presentation of WGS data for iGAS/GAS be improved?

Please select all that apply

- Standardisation of WGS reports (all reports adhere to the same format and contain identical information)
- Clear summary at the start of WGS report stating which samples cluster together/are part of the outbreak   Inclusion of recommendations for investigation of transmission links
- Other, please specify

41. How else could the provision of WGS data for iGAS/GAS be improved?

Section 15: Advantages and disadvantages of replacing emm typing with WGS

In this section we will ask you about your views on the possibility that emm typing will be completely replaced by WGS in the future.

42. Overall, do you think the availability of WGS data (SNP and phylogenetic analysis) rather than just emm typing will have a positive effect on the following aspects?

	Yes, positive effect	No, no effect	No, negative effect	Don’t know
Reliability of data				
Usability of data				
Timeliness of data				
Outbreak management				
Identification of epidemiological links				
Surveillance support				
Overall public health action/ response to iGAS/GAS				

43. If emm typing was to be replaced with WGS, would you see any disadvantages in this?

Please select all that apply

- Complex WGS data or reports are difficult to use for decision making Longer turn-around time for results
- No disadvantages
- Unsure/Don’t know
- Other, please specify

44. Are there any additional uses you envision for WGS data that could help you and/or your team in your work?

- Yes
- No
- Don't know

45. If yes, please describe these potential additional uses of WGS data:

Section 16: Final comments

46. Do you have other comments that may be helpful for this evaluation?
